# Study of the Effects of Remote Heavy Group Vibrations on the Temperature Dependence of Hydride Kinetic Isotope Effects of the NADH/NAD^+^ Model Reactions

**DOI:** 10.1021/acsomega.4c02383

**Published:** 2024-04-24

**Authors:** Grishma Singh, Ava Austin, Mingxuan Bai, Joshua Bradshaw, Blake A. Hammann, Daniel E. K. Kabotso, Yun Lu

**Affiliations:** Department of Chemistry, Southern Illinois University Edwardsville, Edwardsville, Illinois 62026, United States

## Abstract

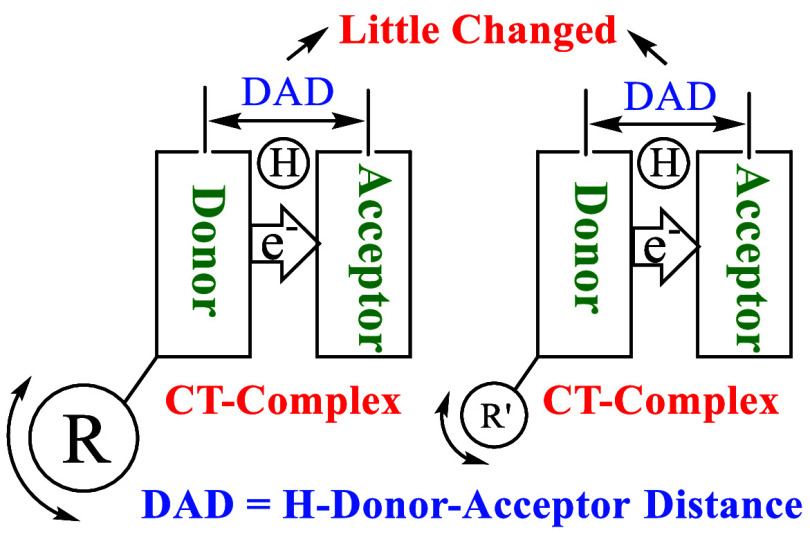

It has recently been observed that the temperature(*T*)-dependence of KIEs in H-tunneling reactions, characterized by isotopic activation energy difference (Δ*E*_a_ = *E*_aD_ – *E*_aH_), is correlated to the rigidity of the tunneling ready states (TRSs) in enzymes. A more rigid system with narrowly distributed H-donor–acceptor distances (DADs) at the TRSs gives rise to a weaker *T*-dependence of KIEs (i.e., a smaller Δ*E*_a_). Theoreticians have attempted to develop new H-tunneling models to explain this, but none has been universally accepted. In order to further understand the observations in enzymes and provide useful data to build new theoretical models, we have studied the electronic and solvent effects on Δ*E*_a_’s for the hydride-tunneling reactions of NADH/NAD^+^ analogues. We found that a tighter charge-transfer (CT) complex system gives rises to a smaller Δ*E*_a_, consistent with the enzyme observations. In this paper, we use the remote heavy group (R) vibrational effects to mediate the system rigidity to study the rigidity−Δ*E*_a_ relationship. The specific hypothesis is that slower vibrations of a heavier remote group would broaden the DAD distributions and increase the Δ*E*_a_ value. Four NADH/NAD^+^ systems were studied in acetonitrile but most of such heavy group vibrations do not appear to significantly increase the Δ*E*_a_. The remote heavy group vibrations in these systems may have not affected the CT complexation rigidity to a degree that can significantly increase the DADs, and further, the Δ*E*_a_ values.

## Introduction

Kinetic isotope effect (KIE) and its temperature (*T*) dependence are important tools to study the hydrogen(H)-transfer reaction mechanisms. Semiclassically, the primary deuterium (D) KIE is smaller than 7, and the isotopic activation energy difference (Δ*E*_a_ = *E*_aD_ – *E*_aH_), which reflects the *T*-dependence of KIEs, is between 1.0 and 1.2 kcal/mol.^1^ When KIEs are outside of the limits, a quantum H-tunneling mechanism is suggested, but discussion of the nature of the tunneling including the structure of the corresponding transition state (TS), i.e., the tunneling-ready state (TRS), has seldom been mentioned.^1^ Traditional Bell tunneling model does not provide any prediction about the relationship between structure and tunneling probability and cannot explain all of the KIE results. In the past three decades or so, there has been evidence showing that many H-transfer reactions that have KIEs even within the semiclassical limits also involve H-tunneling,^2−7^ and some observations are recently explained by models that involve full tunneling mechanisms.^8−14^ These latter tunneling models provide a possibility to understand the relationship between structure and tunneling mechanisms.

One full tunneling model is the vibration-assisted activated H-tunneling (VA-AHT) model.^10,12,13^ This model contains two orthogonal activation processes, in one process, thermal heavy atom motions (vibrations) bring a H-donor (Don-H) and -acceptor (Acc) to the activated TRS ([Don-H---H-Acc]^‡^) that has degenerate reactant [Don-H]^‡^ and product [H-Acc]^‡^ states for H-tunneling ready to occur at a ground state level, and in the other process, the motions sample the shorter donor–acceptor distances (DADs) allowing effective tunneling to happen. (In the TRS, H is at both the donor and acceptor at the same time, being in a quantum state.) Since the first activation process is H-isotope insensitive and the D-tunneling requires shorter DADs due to the shorter wavelength of D vibrations, Δ*E*_a_ results from the second DAD sampling activation process and *E*_aD_ is larger than *E*_aH_ (Δ*E*_a_ > 0). When the system is rigid enough to make the DAD sampling impossible, *E*_aD_ is equal to *E*_aH_ making Δ*E*_a_ = 0. Therefore, a more rigid system gives rise to a smaller Δ*E*_a_. This model predicts a relationship between structure and Δ*E*_a_ magnitudes.

The VA-AHT model has, however, not been universally accepted.^12,15−17^ Test of the model can use the study of the system rigidity−Δ*E*_a_ relationship. Study of the latter relationship could also provide information to help build future necessary H-transfer/tunneling models. A highly rigid system represents a system of densely populated DADs which could be sampled by strong heavy atom vibrations on the DAD sampling coordinate. The rigidity/DAD−Δ*E*_a_ relationship has been studied for H-transfer reactions in enzymes and solution. In wild-type enzymes, KIE has been frequently found to be *T*-independent (Δ*E*_a_ ∼ 0) but become *T*-dependent (Δ*E*_a_ > 0) with mutants.^7−10,13,14,18−31^ It has been found from many studies that DADs are narrowly distributed in wild-type enzymes but they become widely populated in mutants, supporting such a relationship.^11,26,28−30,32^ Results have been used to provide insight into how protein dynamics modulate the short and thus narrowly distributed DADs in enzyme catalysis.

We are the first to systematically investigate the said relationship using the “simpler” hydride transfer reactions in solution, and our results appear to be consistent with the proposed DAD−Δ*E*_a_ relationship as well.^33−39^ In our studies, we use structural and solvent effects to modulate the rigidity of the systems and correlate the DAD information with the observed Δ*E*_a_’s. We use the hydride transfer reactions of NADH/NAD^+^ analogues for the study. One reason to use these reactions is that the hydride transfer takes place in a π–π charge-transfer (CT) complex so that the system rigidity design could use the electronic and steric considerations. Another reason is that the current rigidity/DAD−Δ*E*_a_ relationship study for hydride transfer enzymes only involve NADH/NAD^+^ coenzymes, so that our study can provide useful insight into the explanation of the observations in the corresponding enzymes. We found that a large difference in Δ*E*_a_ comes from systems of very different hydride donating or accepting abilities that give significantly different tightness of the CT complexations and/or from systems of very different donor/acceptor structures that have interactions at various sites leading to different numbers of TRS complexes and thus very different DAD populations.

Another factor that potentially affects the system rigidity and can be used for our study may be the motions/vibrations of the remote heavy groups that have the same electronic properties and same steric effects. It should be noted that the vibrational effects of the heavy enzymes (heavy isotope substituted proteins) on system rigidity and Δ*E*_a_’s have been studied, some of which did give larger Δ*E*_a_ values.^19,40−42^ This has been explained in terms of the slower local vibrations of proteins that lead to broadly distributed DADs. Here, we hypothesize that heavier remote group vibrations would slow down the CT complexation vibrations resulting in wider DAD populations and a larger Δ*E*_a_. We use four NADH/NAD^+^ systems with remote heavy groups (R) designed to investigate the specific hypothesis. These include hydride transfers from 10-ε-alkylated (R) acridines (R–AH, R = methyl, *n*-propyl, and benzyl) to the 9-phenylxanthylium ion (PhXn^+^BF_4_^–^) (System 1), from Hantzsch ester (HEH) to the oxidized forms of the R–AH’s, i.e., 10-ε-alkylated acridinium ions (R–A^+^BF_4_^–^, plus R = *n*-hexyl) (System 2), from isopropanol and its β-deuterated and β-alkylated analogues (R = cyclohexyl (c-HexOH), *n*-propyl (4-HepOH)) to PhXn^+^BF_4_^–^ (System 3), and from 2-phenyl-1,3-dimethylbenzimidazole (DMPBIH) to the R–A^+^BF_4_^–^ (System 4). The *T*-dependence of KIEs of the reactions in acetonitrile were determined. The effects of the remote heavy group vibrations on Δ*E*_a_’s as well as the feasibility of the system design are discussed. It was found that most of these remote groups do not significantly affect the Δ*E*_a_’s, being outside of our expectations. Main factors that affect the Δ*E*_a_ values in these and previously published systems are discussed.
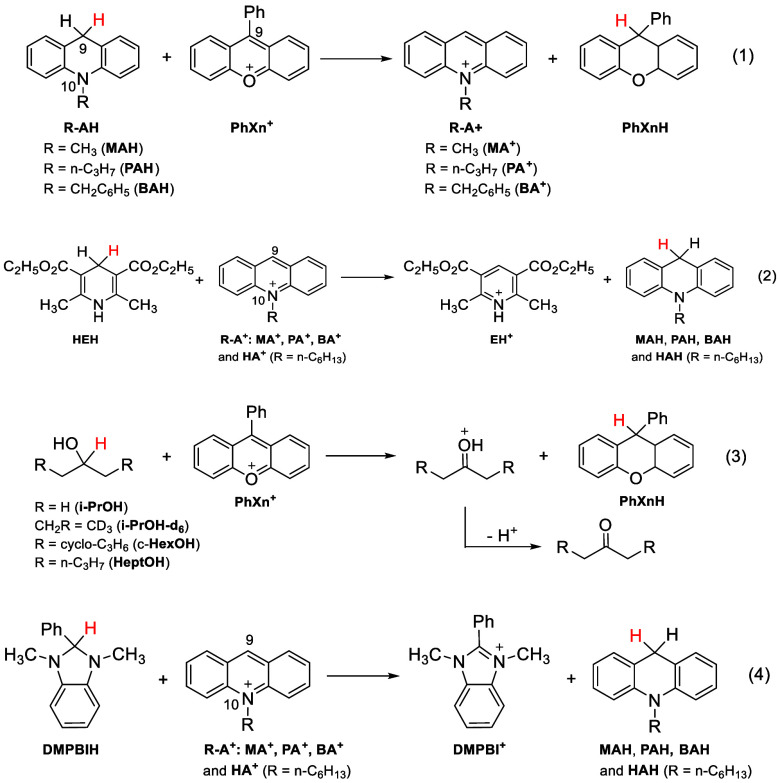


## Results and Discussion

It is well-known that hydride-transfer in the NADH/NAD^+^ model reactions takes place within a CT complex of reactants.^34,43−45^ We call these complexes as productive reactant complexes (PRCs). We have reported the spectroscopy evidence for the CT complex formation in the similar reactions.^34,46^ The PRCs are believed to form in a diffusion-controlled rate. Theoretically, the hydride-transfer could be classical through a transition state (TS) or nonclassical through a TRS. This mechanism is described in eq 5.^37^ The observed KIEs are derived from the second-order rate constants (*k*_2_). They are related to the hydride transfer step (*k*_H_), i.e., KIE = *k*_2H_/*k*_2D_ = *k*_H_/*k*_D_.


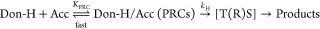
5

Perhaps, the best R group to use for study of the vibrational effects on the DAD sampling would be at an indirect remote reaction center whose hybridization also changes during the reaction and whose vibrations thus likely accompany with vibrations of the direct reaction centers. In the R–AH and R–A^+^, the 10-δ-R groups are at such a N reaction center whose hybridization changes in between sp^2^ and sp^3^ (for systems (1), (2), and (4)) so their vibrations would be expected to affect the DAD sampling. In the β-substituted isopropanol (RCH_2_CH(OH)CH_2_R) systems (3), our study looks into the effects of vibrations of the whole RCH_2_ groups. To study the vibrational effects of the R groups only, the R must at first have very similar, if not the same, electronic effects. Indeed, the R groups we use in this paper have the similar substituent constants: CH_3_ (σ = −0.17), *n*-C_3_H_7_ (σ = −0.13), *n*-C_6_H_13_ (σ = ∼ −0.16), and CH_2_Ph (σ = −0.09), basically satisfying the similar electronic property requirement. Another prerequisite for the study is their minimal steric interactions with the other reactant so that the system flexibility or DAD sampling would not be affected. While all of the R groups are remote from the reaction center so the minimal steric effect requirement is expected to be met, the steric effect is a complex factor that we will discuss for the individual system subsequently.

The representative second-order rate constants (*k*_2_) and KIEs at 25 °C, the enthalpies (Δ*H*^‡^) and entropies (Δ*S*^‡^) of activation, as well as the Δ*E*_a_’s of the reactions are listed in Table 1. According to the reported hydride affinities of the PhXn^+^ (−Δ*G*_H_– = 91.6 kcal/mol), MA^+^ (76.2), HE^+^ (64.4), DMPBI^+^ (49.2) in acetonitrile,^47^ the corresponding reactions are largely exothermic. For example, Δ*G*° = −15.4 kcal/mol for reaction (1) (R = Me), −11.8 kcal/mol for reaction (2) (R = Me), and −27.0 kcal/mol for reaction (4) (R = Me). We did not find the corresponding hydride affinity values of the oxidized forms of the alcohols, but we calculated the Δ*G*° = −4.6 kcal/mol for the hydride-transfer reaction (3) (R = H for isopropanol) in acetonitrile. The latter results indicate that the hydride transfer step of system (4) reactions is the least exothermic, making the reactions the slowest with highest enthalpies of activation among the four series of reactions. All of the entropies of activation are large negative values conforming to the fact that the bimolecular hydride transfer takes place in the tight CT complexes.

**Table 1:** Structural Effects on the *T*-Dependence of Hydride KIEs of the Hydride-Transfer Reactions in Acetonitrile^a^

entries	donor(Don-H)	acceptor^b^(Acc)	*k*_2H_^25 °C^(M^–1^s^–1^)	Δ*H*_H_^‡^(kcal/mol)	Δ*S*_H_^‡^(cal/mol·K)	KIE^25 °C^	Δ*E*_a_(kcal/mol)
System (1)							
1^c^	MAH	PhXn^+^	4.10(0.03) × 10^2^	7.51(0.05)	–21.6(0.2)	4.08 (0.03)	0.88 (0.05)
2	PAH	PhXn^+^	1.43(0.02) × 10^3^	5.70(0.07)	–25.2(0.2)	4.60 (0.05)	0.94 (0.08)
3	BAH	PhXn^+^	3.79(0.02) × 10^2^	6.59(0.03)	–24.8(0.1)	4.26 (0.03)	0.88 (0.05)
System (2)							
4^d^	HEH	MA^+^	1.56(0.01) × 10^2^	5.71(0.03)	–29.5(0.1)	4.92 (0.04)	0.95 (0.10)
5	HEH	PA^+^	1.57(0.01) × 10^2^	5.54(0.04)	–30.1(0.1)	4.92 (0.03)	1.20 (0.10)
6	HEH	HA^+^	1.53(0.00_2_) × 10^2^	5.71(0.03)	–29.6(0.1)	5.02 (0.01)	1.07 (0.06)
7	HEH	BA^+^	5.73(0.03) × 10^2^	4.78(0.12)	–30.0(0.4)	4.53 (0.04)	1.01 (0.12)
System (3)							
8	*i*-PrOH	PhXn^+^	2.02(0.05) × 10^–5^^e^	1.37(0.02) × 10	–34.1(0.6)	3.63 (0.23)^d^	0.83 (0.27)
9	*i*-PrOH-β,β-d_6_	PhXn^+^	2.01(0.05) × 10^–5^^e^	1.34(0.02) × 10	–34.9(0.5)	3.64 (0.16)^d^	0.80 (0.19)
10	c-HexOH	PhXn^+^	2.68(0.01) × 10^–5^^e^	1.35(0.02) × 10	–34.2(0.7)	3.68 (0.16)^d^	0.90 (0.27)
11	4-HepOH	PhXn^+^	5.63(0.01) × 10^–6^^e^	1.36(0.02) × 10	–36.8(0.7)	3.31 (0.06)^d^	0.64 (0.35)
System (4)							
12^c^	DMPBIH	MA^+^	2.12(0.01) × 10^2^	7.20(0.07)	–23.9(0.2)	3.57 (0.03)	0.43 (0.15)
13	DMPBIH	PA^+^	1.28(0.01) × 10^2^	7.07(0.19)	–25.1(0.6)	3.32 (0.04)	–0.02 (0.35)
14	DMPBIH	HA^+^	1.36(0.01) × 10^2^	7.23(0.18)	–24.5(0.6)	3.39 (0.03)	–0.04 (0.30)
15	DMPBIH	BA^+^	5.78(0.04) × 10^2^	6.36(0.09)	–24.6(0.3)	3.10 (0.04)	0.40 (0.22)

a Numbers in paratheses are standard deviations.

b Counterion: BF_4_^–^.

c From ref^35^

d From ref^34^

e For 22 °C.

Like other hydride transfer reactions of NADH/NAD^+^ models, these reactions have small KIEs (<7). The Δ*E*_a_’s are from ∼ 0–1.11 kcal/mol, some of which are within and some of which are outside of the semiclassical range of 1.0–1.2 kcal/mol. While such hydride transfer reactions of NADH/NAD^+^ analogues usually have small KIEs, both this and other works of ours as well as a few sporadic work from others showed that they have Δ*E*_a_’s spanning a wide range from well below the semiclassical limit (close to 0 kcal/mol), through the semiclassical range, to well above the semiclassical limit (up to ∼1.8 kcal/mol).^33−35,46,48^ Furthermore, it has been shown that small KIEs from such hydride transfer reactions also fit to the Marcus theory of atom transfer that involves a H-tunneling component.^2,4,5^ In the meantime, the small KIE’s and similar Δ*E*_a_ values were also found in the hydride transfer reactions of NADH/NAD^+^ in enzymes and mutants.^11,13,25,26,28,49,50^ As described in the Introduction, the latter observations have been explained following contemporary H-tunneling models.

### The Remote ε-R Group Effects on Δ*E*_a_ in Systems (1) and (2) Reactions

The ultimate goal of the work is to correlate the system rigidity with Δ*E*_a_. The overall hypothesis of our group study is that a more rigid system gives rise to a smaller Δ*E*_a_ value. *The specific hypothesis in this paper is that slower vibrations of a heavier remote group increase the DAD sampling range and thus the ΔE*_*a*_*value*. Table 1 shows, however, that the change of R groups does not significantly change the Δ*E*_a_ in most of the systems. The Δ*E*_a_’s are in the range from 0.88 to 0.94 kcal/mol for the reactions between R–AH and PhXn^+^ (System 1), and in the range from 0.95 to 1.20 kcal/mol for the reactions between RA^+^ and HEH (System 2). A positive observation in these two systems ((1) and (2)) is that none of the Δ*E*_a_ values are smaller than those for the reactions with the lightest remote CH_3_ group (MAH and MA^+^), but use of the relatively small Δ*E*_a_ differences between reactions of different remote R groups to support our specific hypothesis in this paper may be reluctant. An interesting observation is, however, that the reactions of PAH and PA^+^ (R = *n*-C_3_H_7_) with the corresponding acceptor (PhXn^+^) and donor (HEH) have consistently relatively larger Δ*E*_a_ than those with MAH and MA^+^ (*R* = CH_3_). Using the reactions of these four compounds (PAH vs MAH, and PA^+^ vs MA^+^) with other hydride acceptors/donors to investigate the specific hypothesis in this paper is currently in progress to attempt to find whether the trend found is consistent throughout a large range of the reactions.

### The Remote β-R Group Effects on Δ*E*_a_ in System (3) Reactions

The remote R group effects on Δ*E*_a_’s for the System (3) reactions are largely the same as those for the systems (1) and (2), i.e., less significant effects were observed. The Δ*E*_a_ values have much larger deviations (different ways to determine the slow kinetics from others, see the Experimental and Computations section). This system does not have a π–π but an n−π complexation between alcohol O and the PhXn^+^ ring, according to our previous report, so that the complexation vibrations could also affect the DAD sampling.^51,52^ Note that we have reported the Δ*E*_a_ value (1.01 ± 0.26 kcal/mol) for the reaction of primary benzyl alcohol with PhXn^+^ in acetonitrile.^33^ It is close to the Δ*E*_a_ values of the reactions of the secondary alcohols in Table 1 (mostly 0.8 – 0.9 kcal/mol), further indicating that the alcohol group effect on the Δ*E*_a_’s of the class of reactions is small. Herein, change of the two CH_3_ groups in isopropanol to two heavier CD_3_ groups of the same electronic and steric effects (entries 8 vs 9 in Table 1) increases their mass by 20% but the Δ*E*_a_ has almost no change. Note that we are aware that the β,β-2CH_3_/2CD_3_ secondary (2°) KIE may affect the *T*-dependence of observed 1° KIEs, but it is very small, which is 1.05 at 25 °C, as we reported.^51,53^ Therefore, *T*-dependence of such small 2° KIEs would not significantly affect the Δ*E*_a_ value. The observed same Δ*E*_a_ values from the two reactions suggest that the slower CD_3_ vibrations do not appear to broaden the DAD populations in the reaction of isopropanol.

**Figure 1:**
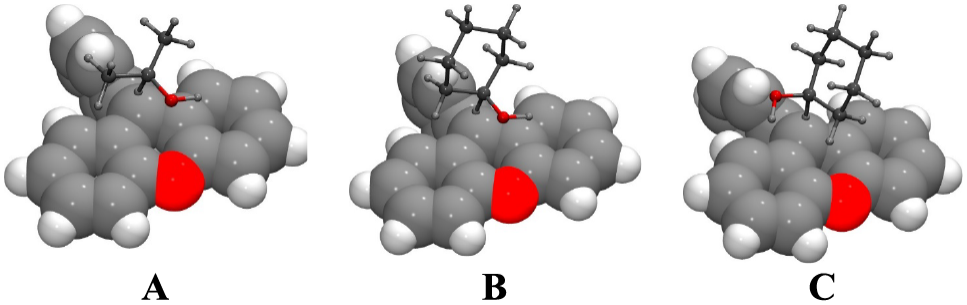
Most populated gas-phase TS structures for the reactions of PhXn^+^ with isopropanol (**A**, one of the three alcohols found, accounting for 94% of all) and cyclohexanol (**B** and **C**, two of the eight alcohols found, accounting for 83% of all). The space-filling structures in the background represent PhXn^+^, and the ball-and-stick structures in the foreground represent alcohols. The red atom is O.

To compare the vibrational effects of the β-R groups in isopropanol (R = H) vs cyclohexanol (R = −CH_2_–CH_2_–CH_2_−) on Δ*E*_a_ values (entries 8 vs 10), their steric effect difference should be discussed as they are relatively closer to the reaction center as compared to the ε-R group effects in systems (1) and (2). From both the classical TS and nonclassical TRS structures of the isopropanol reaction we reported, the 9-phenyl group of the PhXn^+^ is far from the two alcohol methyl groups due to the restricted geometry of the T(R)S in which the transferring hydride points toward the 9-C of the PhXn^+^ and the alcohol O complexes with the central ring of the same (also cf. the subsequent Figure 1 (**A**)).^51,52,54^ Change of the two methyl groups in isopropanol to the cyclic hexyl group in cyclohexanol would be expected to make the steric effect little changed. To confirm the latter, we calculated the classical TS structures for both reactions in the gas phase. (We regard that the TRS structure has the similar geometry as, or close to, the classical TS, except for the distance between the donor/acceptor carbons.)^36,38,52,54^ The most populated TS structures of the reactions of isopropanol (structure **A**) and cyclohexanol (**B** and **C**) are shown in Figure 1. From these structures, we found that the 3,4,5-CH_2_–CH_2_–CH_2_– group in cyclohexanol do lead away from the 9-phenyl group of the PhXn moiety. Therefore, the difference of the Δ*E*_a_ values between the reactions of these two alcohols reflect largely the difference of the vibrational effects of the R groups. Due to the large standard deviations in Δ*E*_a_ values of the two reactions, however, the remote R group effect on Δ*E*_a_ cannot be differentiated.

The Δ*E*_a_ for the reaction of 4-heptanol (entry 11) is the smallest among the System (3) reactions, but again the error is large so that its difference from that for the reaction of isopropanol may not give enough evidence to support the specific hypothesis in this paper. If this difference is real; however, it might be that the free rotation of the two large CH_3_CH_2_CH_2_– groups interact with the 9-phenyl group of the PhXn more often making the system more rigid and decreasing the Δ*E*_a_ value as compared to that of the reaction of isopropanol. Interestingly, the observed activation entropy (Δ*S*^‡^) of this reaction is the most negative value among the reactions of four alcohols, implicating that the system has a very tight reactive complex. Overall, the results from the System (3) reactions do not provide evident support for the specific hypothesis in this paper that relates remote heavy group vibrations to the Δ*E*_a_ values.

### The Remote ε-R Group Effects on Δ*E*_a_ in System (4) Reactions

The System (4) reactions give us unexpected Δ*E*_a_ results from changes of the R group in the acceptor of R–A^+^. It should be mentioned first that the Δ*E*_a_ of the reaction of DMPBIH with MA^+^ is much smaller than that of the reaction of HEH with the same (0.27 vs 0.95 kcal/mol in Table 1). We have found that the productive reactant complexes (PRCs) of the former reaction are tighter than those of the latter.^34^ That is possibly due to the fact that the DMPBIH is a 16.2 kcal/mol stronger hydride donor than the HEH so that the CT complexation is tighter in the former than the latter.^47^ Here, in the System (4) reactions using DMPBIH as a donor, when the size of the R group in the acceptors R–A^+^ increases from methyl (for MA^+^) to propyl (for PA^+^) and hexyl (for HA^+^), the KIE becomes almost *T*-independent (Δ*E*_a_ from 0.27 to ∼ 0 kcal/mol, in Table 1) (also see Figures S1, S2). This significant decrease of the Δ*E*_a_ value, rather than increase as expected, is possibly due to the steric interaction of the R group with the benzene ring fused with the 1,2-dihydroimidazoline ring of DMPBIH so that the system rigidity increases as the R size increases. We have reported the PRC structures of the reaction of DMPBIH with MA^+^ and found that the most populated PRC structure has the CH_3_ group in MA^+^ being “stuck” in between the “fused benzene” ring and one N–CH_3_ group of the DMPBIH (cf. Figure 2 of ref^38^). (This steric effect caused by the “remote” benzene structure of the DMPBIH is that the other donors in this work do not have.) It can thus be imagined that increase of the size of the R group would increase the rigidity of the system. Therefore, the significant decrease of the Δ*E*_a_ due to the R change from methyl to the bulkier propyl or hexyl is likely resulted from the system rigidity increase due to the augmenting steric interactions between the donor and acceptor. One would indicate that the standard deviations in the Δ*E*_a_ values are relatively large in these latter two systems so that the explanation may lack strong support, but that both systems have the same behaviors of almost *T*-independence of KIEs would suggest that the difference could be true (see Figures S1,S2). As far as the reaction of BA^+^ is concerned, the Δ*E*_a_ is the same as that of the reaction of MA^+^ within the experiment error. We do not have a good explanation for this result but the benzyl group in BA^+^ may also interact with the aromatic structures of the DMPBIH through π–π interaction altering the system rigidity. Nonetheless, we did not see the remote R vibrational effects on Δ*E*_a_ in this series of reactions in a way to support the specific hypothesis in this work.

### The Remote R Group Effects on *k*_2_

The remote heavy group effects on the rates (*k*_2_) of the reactions refuse to be generalized among the four systems (Table 1). In System (1), *k*_2_(PAH) > *k*_2_(MAH) ∼ *k*_2_(BAH). In System (2), *k*_2_(BA^+^) > *k*_2_(MA^+^) ∼ *k*_2_(PA^+^) ∼ *k*_2_(HA^+^). The observed smallest enthalpy of activation of the corresponding reactions of PAH and BA^+^ are mainly responsible for their fastest rates in the respective series of reactions (compare Δ*H*^‡^ values in Table 1). This is the same for the observed fastest reaction of BA^+^ with DMPBIH among the System (4) reactions. In the rest of the System (4) reactions, *k*_2_(MA^+^) > *k*_2_(PA^+^) ∼ *k*_2_(HA^+^). The observed more negative entropies of activation (compare Δ*S*^‡^ values in Table 1) for the reactions of PA^+^ and HA^+^ largely contribute to their slower rates. Lastly, in System (3), *k*_2_(isopropanol) ∼ *k*_2_(isopropanol-β,β-2d) ∼ *k*_2_(c-HexOH) > *k*_2_(4-HepOH). The observed slowest reaction of 4-HepOH is likely mainly resulted from the observed most negative Δ*S*^‡^ value among the four reactions.

## Conclusions

Our group is the first to systematically study the structural effect on the *T*-dependence of KIEs (represented by Δ*E*_a_ values) for the hydride transfer reactions in solution. Our overall hypothesis on the basis of the enzymatic observations and explanations is that a more rigid system with densely populated short DADs in H-tunneling reactions gives rise to a smaller Δ*E*_a_ value. While the rigidity(DAD)−Δ*E*_a_ relationship has been studied in enzymes, in our research, we use the hydride tunneling reactions of NADH/NAD^+^ coenzyme analogues to investigate the relationship in our hypothesis. Structural (electronic and steric) effects as well as solvent effects (including polarity and protic/aprotic considerations) on the Δ*E*_a_’s of the hydride transfer reactions have been studied. Results appear to be consistent with the enzymatic observations and support our hypothesis.

Another factor that can potentially affect the system rigidity is the structural vibrations. In this paper, we chose the remote heavy group vibrational effects to study. The remote groups chosen have the similar electronic effects and cause little steric effects so that the vibrational effects could be isolated to study. The remote groups are connected to the remote indirect reaction centers whose vibrations likely couple to the reaction center vibrations. *The specific hypothesis of this paper is that slower vibrations of a heavier remote group increase the DAD sampling range and thus increase the ΔE*_*a*_*value.* We designed the systems that contain such remote groups and determined the Δ*E*_a_’s in acetonitrile. We found that the remote heavy groups do not generally significantly increase the Δ*E*_a_’s in systems (1)–(3) where the steric effect appears not to be an issue. Importantly, none of these systems show that these groups decrease the Δ*E*_a_ values as compared to the lightest methylated counterparts. This made us to infer that the remote heavy groups may have increased the Δ*E*_a_’s, i.e., consistent with the specific hypothesis. Therefore, even if the increases are so small that most of them fall within the experimental errors in these systems, we regard that it is worth to continue to investigate the remote group vibrational effect on the Δ*E*_a_’s for a large range of reactions or for other types of H-transfer reactions.

While our results appear not to provide strong support for the specific hypothesis we proposed in this paper, they, together with our previously published results from the structural and solvent effects study, suggest that the strength of the CT complexations due to the electronic properties of the donor and acceptor largely determine the system rigidities and Δ*E*_a_ values. That is, the stronger CT complexations of more densely populated DADs, which are favored by stronger electron donors/acceptors, give rise to a smaller Δ*E*_a_ value. In enzymes, study of the relationship uses different enzyme structures, rather than different substrate structures from our work; therefore, the DAD sampling difference is largely caused by the difference in protein vibrations. Nonetheless, the growing body of our results will be valuable addition to the current debates on the appropriateness of theories to describe hydride- as well as general H-tunneling reactions. They could also provide insight into the contentious role of protein dynamics in DAD sampling activation and enzyme catalysis.

## Experimental and Computations

### General Procedures

Syntheses of the following compounds were previously reported from our group: 10-methylacridinium ion (MA^+^BF_4_^–^), 9-phenylxanthylium ion (PhXn^+^BF_4_^–^), 10-methyl-9,10-dihydroacrdine (MAH) and its 9,9’-dideuterated derivative (MA-H-9,9’-d,d), Hantzsch ester (diethyl 1,4-dihydro-2,6-dimethyl-3,5-pyridinedicarboxylate, HEH) and its 4,4’-dideuterated derivative (HEH-4,4’-d,d), and 1,2-dimehtyl-2-phenyl-1,2-dihydrobenzimidazoline (DMPBIH) and its 2-deuterated derivative (DMPBID).^34,51,55^ The 10-propylacridinium ion (PA^+^I^–^) was synthesized by reacting acridine with 1-iodopropane in acetonitrile in a high-pressure reaction vessel at 120 °C for 3 days. The 10-hexylacridinium ion (HA^+^I^–^) and 10-benzylacridinium ion (BA^+^Br^–^) were synthesized by reacting acridine with 1-iodohexane and benzyl bromide, respectively, at 130 °C for 30 min. The reduced forms (PAH, HAH, and BAH) of these salts/cations were prepared by reduction of the above salts with NaBH_4_. Their deuterated derivatives (PAH-9,9’-d,d and BAH-9,9’-d,d) were synthesized from the reduction of the corresponding 9-acridones, which were prepared from the oxidation of the corresponding salts by KO_2_, using the method that we used to synthesize the MAH-9,9’-d,d.^51^ Syntheses of the BF_4_^–^ salts of the PA^+^, HA^+^, and BA^+^ are from the reactions of PAH, HAH, and BAH with tropylium tetrafluoroborate (Tr^+^BF_4_^–^) using a procedure of ours.^55^ Synthesis of 2-propanol-β-d_6_ ((CD_3_)_2_CHOH), 2-propanol-α-d ((CH_3_)_2_CDOH), and 2-propanol-α-d-β-d_6_ ((CD_3_)_2_CDOH) were reported previously from our group.^51,56^ Other normal alcohol hydride donors were purchased and redistilled before use. Their α-deuterated alcohols were synthesized by reduction of the corresponding ketones (commercially available) by NaBH_4_ or NaBD_4_ using the same procedure as used for the synthesis of the above deuterated isopropanol (See Supporting Information). The D content in all deuterated compounds are >98% (by NMR). The HPLC grade acetonitrile was redistilled twice under nitrogen, with the presence of KMnO_4_/K_2_CO_3_ (to remove the reducing impurity) and P_2_O_5_ (to remove water) in order, for kinetic measurements.

### Kinetic Measurements of the Systems (1), (2), and (4) Reactions

Kinetics of these reactions were determined on the SF-61DX2 Hi-Tech KinetAsyst double-mixing stopped-flow instrument. Same kinetic procedures in our publications were followed.^34,35,37^ From our experiments and literature, the type of reactions strictly follow the second-order rate law.^4,33−35,47,55,57^ Each KIE was derived from the second-order rate constants of the isotopic reactions (=*k*_2H_/*k*_2D_). Experimentally, the pseudo-first order rate constants (*k*^pfo^’s) were determined spectroscopically (by UV–vis) and the observed *k*_2_ was calculated from dividing *k*^pfo^ by the concentration of the large excess substrate (Sub) (for example, Sub-H or Sub-D), i.e., *k*_2_ = *k*^pfo^/[Sub-H(D)]. Then,


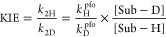
6

Usually, the same concentrations of Sub-H and Sub-D solutions were used.

Six measurements of *k*^pfo^’s for the reactions of two isotopologues at different temperatures were made on the same day and repeated on other day(s). For a Δ*E*_a_ determination, kinetics was determined over a temperature range of 40 °C, and the *E*_aH_ and *E*_aD_ were derived, respectively. A typical kinetic procedure at certain temperature is as follows. Six kinetic runs of 12 half-lives of the reaction were measured for each isotopic reaction back-to-back. The procedure was then repeated at other temperatures as quickly as possible (for example, 5, 15, 25, 35, and 45 °C, in order) so that the instrument settings were kept the same and the aging of the reaction solutions was the minimum (while the solutions were already stable, they were wrapped with aluminum foil and kept in refrigerator between temperatures to eliminate any possible error source.). Repetitions or kinetic measurements of the reactions of the same series of substituted substrates on different days sometimes used different batches of substrates and solvents and sometimes were done by different workers. That was to eliminate the effect of possible different impurity from unknown sources or human errors on the KIE measurements. Therefore, one KIE value was obtained from 18 repetitions. Pooled standard deviations were reported. Kinetic results (from the extent of reaction of close to 1% to 99.98% (corresponding to 12 half-lives)) were fitted very well/excellently to the first-order rate law for *k*^pfo^ derivation and to the Arrhenius correlations for *E*_a_ derivation, both with *R*^2^ = 0.9990–1.0000, many closer or sometimes even equal to 1.0000! Other details about the kinetic measurements as well as the raw data can be found from Tables S1–S5 and S10–S12 and the footnotes therein.

### Kinetic Measurements of the System (3) Reactions

Kinetics of these reactions were determined differently from the above procedures. Same procedures in our publications for the study of the class of hydride-transfer reactions were followed.^33,56,58^ The *k*^pfo^ was determined by following the decay of the PhXn^+^ spectroscopically. The *k*_2_ value was calculated from *k*^pfo^/[alcohol], and the KIE was calculated from the *k*_2_ values (= *k*_2H_/*k*_2D_).

80 μL of 0.1 M stock solution of PhXn^+^ in acetonitrile was added to 8.0 mL of acetonitrile solution containing large excess of alcohol in a sealed 10 mL reaction vial that was preplaced in a water bath with a desired temperature. About 0.2 mL of the reaction aliquots were periodically taken into sample vials precooled in ice. The samples were immediately placed in a freezer (∼−20 °C) until 6 to 8 reaction aliquots within 1–3 half-lives of the reaction were collected. The aliquots were then analyzed by dilution of a preset volume in acetonitrile containing 3 M HClO_4_, and the corresponding UV–vis spectra at different reaction times, i.e., the kinetic scans, were obtained. Absorbance (Abs) decrease with time at 373 nm due to the PhXn^+^ absorption was recorded. The obtained Abs-*t* data were fit to the first-order rate equation, -ln(Abs) = *k*^pfo^·*t* + constant, and the slope of the linear plot of −ln(Abs) vs *t* was taken as the *k*^pfo^ of the reaction. The linear plots usually have regression coefficients (*R*^2^) greater than 0.995. Each kinetic run was determined more than 2 times in most cases (see Tables S6–S9). Parallel determinations of the rates of the reactions involving normal and deuterated alcohols of same concentrations were used to derive the KIEs = (*k*_2H_*/k*_2D_ = *k*^pfo^*/k*^pfo^). Other details about the kinetic measurements as well as the raw data can be found from Tables S6–S9 and the footnotes therein.

### Computations

All of the geometries in this work were optimized under the M06–2X^59^/Def2SVP^60^ level of theory with a fine DFT integration grid in Gaussian 09 software. A scaling factor of 0.9687, which was fitted against the ZPVE15/10 database,^61^ was applied in all of the free energy calculations in order to overcome the overestimate nature of the harmonic model. The free energy (Δ*G*°) of the hydride-transfer reaction from isopropanol to PhXn^+^ to generate the protonated acetone and PhXnH in acetonitrile was calculated by using eq 7:
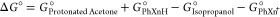
7

*G*°s in the right side of the equation refer to the individual molecules of the reaction. The universal solvation model (SMD) was used.

The percentage (*A*_*i*_) of the gas-phase TS structures of the reactions of isopropanol and cyclohexanol with PhXn^+^ were calculated under the law of Boltzmann distribution of its free energy (*G*_*i*_) by using eq 8:
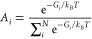
8

In this equation, *N* is the number of the TSs found, *k*_B_ is the Boltzmann constant, and *T* is temperature.

## Supporting Information Available

The exemplified Arrhenius plots of 1° KIE’s, the kinetic rates and KIE data, organic synthesis, as well as the atom coordinates, electronic energies, free energies of the computed ground-state reactants and products, and classical TS’s (PDF)
