# Supplementary material for: Study of the Effects of Remote Heavy Group Vibrations on the Temperature Dependence of Hydride Kinetic Isotope Effects of the NADH/NAD+ Model Reactions
Source: ACS Omega. 2024 Apr 24;9(18):20593–600. doi: 10.1021/acsomega.4c02383 (PMC11080011; doi:10.1021/acsomega.4c02383)
Supplement: Supplementary file 1 — ao4c02383_si_001.pdf [file ao4c02383_si_001.pdf]

Supporting Information

Study of the Effects of Remote Heavy Group Vibrations on the Temperature Dependence of Hydride Kinetic Isotope Effects of the NADH/NAD^+ Model Reactions

Grishma Singh†, Ava Austin†, Mingxuan Bai†, Joshua Bradshaw†, Blake A. Hammann†, Daniel E. K. Kabotso†,‡, Yun Lu†*

† Department of Chemistry, Southern Illinois University Edwardsville, Edwardsville, Illinois 62026, United States

‡ Current address: Department of Basic Sciences, School of Basic and Biomedical Sciences, University of Health and Allied Sciences, PMB 31, Ho, Volta Region, Ghana

yulu@siue.edu

Exemplified Arrhenius plots of KIEs (Figures S1 and S2)………………...S1
Kinetic rates and KIEs (Tables S1 – S12) …...…………………………..……………….............. S2
Organic synthesis …..…………………………………………………………………………. S5
Energies and Atom coordinates for the individual molecules in acetonitrile (SMD model)…………....S7
Energies and Atom coordinates for the TSes of isopropanol and $PhXn^+$ systems in gas phase………...S10
Energies and Atom coordinates for the TSes of cyclohexanol and $PhXn^+$ systems in gas phase……... S12
References ………………………………………………………………………………………...S21

Exemplified Arrhenius plots of KIEs

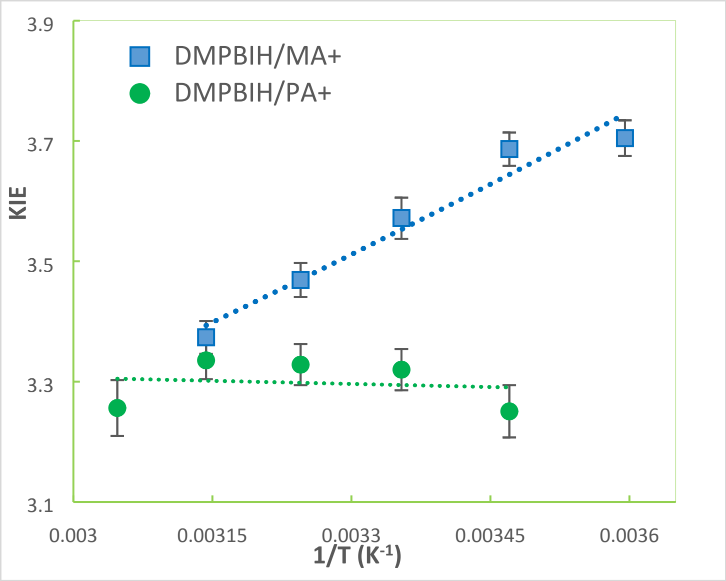

Figure S1. Comparison of the Arrhenius plots of KIEs for the hydride-transfer reaction from DMPBIH to MA^+ (5 to 45 °C) vs. PA^+ (15 to 55 °C) in acetonitrile. Lines are the nonlinear exponential fit of the experimental T – KIE data in Table S10.

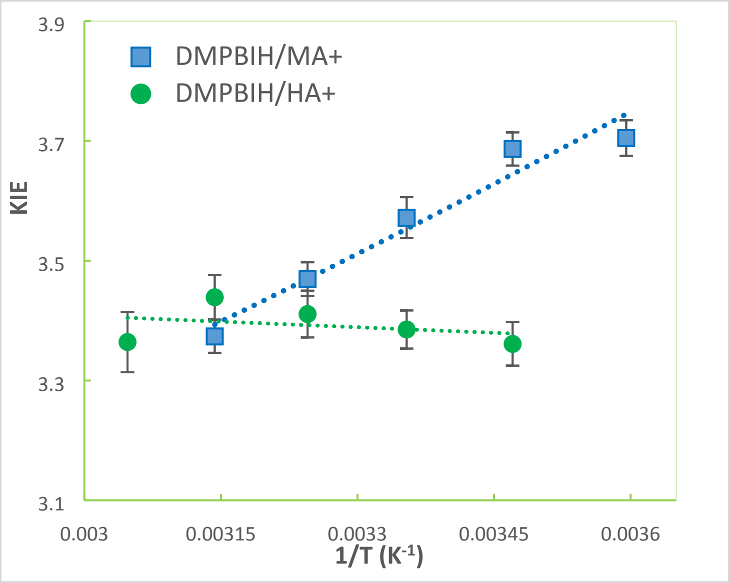

Figure S2. Comparison of the Arrhenius plots of KIEs for the hydride-transfer reaction from DMPBIH to MA^+ (5 to 45 °C) vs. HA^+ (15 to 55 °C) in acetonitrile. Lines are the nonlinear exponential fit of the experimental T – KIE data in Table S11.

Table S1. The temperature effects on the rate constants and 1° KIEs of the hydride transfer reaction from PAH to $PhXn^{+}BF_4^{-}$ in acetonitrile [a,b]

Temp (°C)	k_{2H} ($M^{-1}s^{-1}$)	k_{2D} ($M^{-1}s^{-1}$)	1° KIE
45.0	2.74(0.03)x10^3	6.77(0.04)x10^2	4.04(0.04)
35.0	1.99(0.01)x10^3	4.63(0.04)x10^2	4.31(0.05)
25.0	1.43(0.01)x10^3	3.10(0.04)x10^2	4.60(0.06)
15.0	9.80(0.05)x10^2	2.04(0.01)x10^2	4.81(0.04)
5.0	6.58(0.05)x10^2	1.31(0.01)x10^2	5.01(0.05)

[a] Repeated on three different days with 6 repetitions each day. Numbers in parentheses are the pooled standard deviations S(pooled). [b] [PAH] = 0.006 M, [$PhXn^+$] = 0.0004 M. Absorbance decay at 473 nm due to $PhXn^+$ was followed for kinetic measurements.

Table S2. The temperature effects on the rate constants and 1° KIEs of the hydride transfer reaction from BAH to $PhXn^{+}BF_4^{-}$ in acetonitrile [a,b]

Temp (°C)	k_{2H} ($M^{-1}s^{-1}$)	k_{2D} ($M^{-1}s^{-1}$)	1° KIE
45.0	8.13(0.06)x10^2	2.08(0.02)x10^2	3.90(0.04)
35.0	5.64(0.04)x10^2	1.37(0.01)x10^2	4.12(0.05)
25.0	3.79(0.02)x10^2	8.90(0.04)x10	4.26(0.03)
15.0	2.50(0.01)x10^2	5.52(0.02)x10	4.53(0.03)
5.0	1.60(0.01)x10^2	3.35(0.01)x10	4.78(0.04)

[a] Repeated on three different days with 6 repetitions each day. Numbers in parentheses are the pooled standard deviations S(pooled). [b] [BAH] = 0.003 M, [$PhXn^+$] = 0.0002 M. Absorbance decay at 473 nm due to $PhXn^+$ was followed for kinetic measurements.

Table S3. The temperature effects on the rate constants and 1° KIEs of the hydride transfer reaction from HEH to $PA^{+}BF_4^{-}$ in acetonitrile [a,b]

Temp (°C)	k_{2H} ($M^{-1}s^{-1}$)	k_{2D} ($M^{-1}s^{-1}$)	1° KIE
45.0	3.02(0.03)x10^2	6.93(0.06)x10	4.35(0.06)
35.0	2.15(0.02)x10^2	4.76(0.03)x10	4.52(0.05)
25.0	1.57(0.01)x10^2	3.19(0.01)x10	4.92(0.03)
15.0	1.10(0.01)x10^2	2.09(0.01)x10	5.24(0.04)
5.0	7.52(0.01)x10	1.33(0.01)x10	5.67(0.07)

[a] Repeated on three different days with 6 repetitions each day. Numbers in parentheses are the pooled standard deviations S(pooled). [b] [HEH] = 0.0045 M, [PA^+] = 0.0003 M. Absorbance decay at 436 nm due to PA^+ was followed for kinetic measurements.

Table S4. The temperature effects on the rate constants and 1° KIEs of the hydride transfer reaction from HEH to $HA^{+}BF_4^{-}$ in acetonitrile [a,b]

Temp (°C)	k_{2H} ($M^{-1}s^{-1}$)	k_{2D} ($M^{-1}s^{-1}$)	1° KIE [c]
45.0	3.00(0.01)x10^2	6.59(0.04)x10	4.56(0.01)
35.0	2.15(0.00_4)x10^2	4.55(0.03)x10	4.73(0.01)
25.0	1.53(0.00_2)x10^2	3.06(0.02)x10	5.02(0.01)
15.0	1.06(0.00_1)x10^2	1.99(0.01)x10	5.33(0.01)
5.0	7.19(0.01)x10	1.24(0.01)x10	5.82(0.01)

[a] Repeated on three different days with 6 repetitions each day. Numbers in parentheses are the pooled standard deviations S(pooled). [b] [HEH] = 0.0045 M, [HA^+] = 0.0003 M. Absorbance decay at 436 nm due to HA^+ was followed for kinetic measurements; c The standard deviations for these KIEs are relatively small.

Table S5. The temperature effects on the rate constants and 1° KIEs of the hydride transfer reaction from HEH to $BA^+BF_4^-$ in acetonitrile [a,b]

**Temp (°C)**	k_{2H} **($M^{-1}s^{-1}$)**	k_{2D} **($M^{-1}s^{-1}$)**	**1° KIE**
45.0	1.03(0.01)x10^3	2.51(0.06)x10^2	4.11(0.10)
35.0	7.81(0.06)x10^2	1.79(0.01)x10^2	4.37(0.04)
25.0	5.73(0.03)x10^2	1.26(0.01)x10^2	4.53(0.04)
15.0	4.28(0.02)x10^2	8.69(0.05)x10	4.93(0.04)
5.0	3.04(0.01)x10^2	5.89(0.02)x10	5.16(0.03)

[a] Repeated on three different days with 6 repetitions each day. Numbers in parentheses are the pooled standard deviations S(pooled). [b] [HEH] = 0.0045 M, [BA^+] = 0.0003 M. Absorbance decay at 436 nm due to BA^+ was followed for kinetic measurements.

Table S6. The temperature effects on the rate constants and 1° KIEs of the hydride transfer reaction from *i*-PrOH to $PhXn^+BF_4^-$ in acetonitrile [a,b]

**Temp (°C)**	k_{2H} **($M^{-1}s^{-1}$)**	No. of Measurements	k_{2D} **($M^{-1}s^{-1}$)**	No. of Measurements	**1° KIE**
67.0	4.90(0.15)x10^{-4}	4	1.62(0.04)x10^{-4}	4	3.03(0.12)
60.0	3.12(0.06)x10^{-4}	4	9.89(0.49)x10^{-5}	4	3.15(0.17)
54.0	2.21x10^{-4} [c]	2	6.86x10^{-5} [c]	2	3.22 [c]
45.0	1.19(0.01)x10^{-4}	6	3.40(0.02)x10^{-5}	6	3.49(0.28)
36.0	6.22(0.22)x10^{-5}	4	1.79(0.02)x10^{-5}	3	3.48(0.13)
29.0	3.32x10^{-5} [c]	2	9.13x10^{-6} [c]	2	3.64 [c]
22.0	2.02(0.05)x10^{-5}	4	5.57(0.32)x10^{-6}	4	3.63(0.23)

[a] [*i*-PrOH] = 3.27x10^{-1} M, [$PhXn^+$] = 9.66 x 10^{-4} M. For absorbance measurement, 80 microliters of reaction solution at different times was injected into 1.92mL acetonitrile containing 0.80M HClO4. Absorbance decay at 473 nm due to $PhXn^+$ with time was collected to derive the k^{pfo}; [b] Numbers in parentheses are the pooled standard deviations S(pooled), except otherwise noted; [c] Two times of measurements, within 0.2% error in rates.

Table S7. The temperature effects on the rate constants and 1° KIEs of the hydride transfer reaction from *i*-PrOH-β,β-d_6 to $PhXn^+BF_4^-$ in acetonitrile [a,b]

**Temp (°C)**	k_{2H} **($M^{-1}s^{-1}$)**	No. of Measurements	k_{2D} **($M^{-1}s^{-1}$)**	No. of Measurements	**1° KIE**
67.0	4.74(0.10)x10^{-4}	6	1.57(0.03)x10^{-4}	5	3.03(0.08)
60.0	2.89(0.06)x10^{-4}	4	9.75(0.13)x10^{-5}	4	2.96(0.07)
54.0	2.14x10^{-4} [c]	2	6.57x10^{-5} [c]	2	3.26 [c]
45.0	1.10(0.05)x10^{-4}	4	3.44(0.10)x10^{-5}	4	3.21(0.16)
36.0	5.93(0.08)x10^{-5}	4	1.67(0.15)x10^{-5}	4	3.56(0.33)
29.0	3.38(0.14)x10^{-5}	4	1.02(0.03)x10^{-5}	4	3.32(0.17)
22.0	2.01(0.05)x10^{-5}	4	5.53(0.19)x10^{-6}	4	3.64(0.16)

[a] [*i*-PrOH] = 3.25x10^{-1} M, [$PhXn^+$] = 9.66 x 10^{-4} M. For analysis, 80 microliters of reaction solution at different times was injected into 1.92mL acetonitrile containing 0.80M HClO4. Absorbance decay at 473 nm due to $PhXn^+$ with time was determined for kinetic measurements; [b] Numbers in parentheses are the pooled standard deviations S(pooled), except otherwise noted; [c] Two times of measurements, 0.2% error in rates.

Table S8. The temperature effects on the rate constants and 1° KIEs of the hydride transfer reaction from cyclohexanol (c-HexOH) to $PhXn^+BF_4^-$ in acetonitrile [a,b]

Temp (°C)	k_{2H} ($M^{-1}s^{-1}$)	No. of Measurements	k_{2D} ($M^{-1}s^{-1}$)	No. of Measurements	1° KIE
67.0	6.12(0.43)x10^{-4}	4	2.04(0.13)x10^{-4}	3	2.99(0.28)
60.0	4.11(0.30)x10^{-4}	3	1.33x10^{-5}	2	3.08(0.23)
54.0	2.83x10^{-4} [c]	2	8.71x10^{-5} [c]	2	3.25 [c]
45.0	1.50(0.04)x10^{-4}	4	4.46(0.25)x10^{-5}	4	3.35(0.02)
36.0	8.33x10^{-5} [c]	2	2.35x10^{-5} [c]	2	3.55 [c]
29.0	4.32(0.07)x10^{-5}	3	1.23(0.09)x10^{-5}	4	3.53(0.25)
22.0	2.68x10^{-5} [c]	2	7.28x10^{-6} [c]	2	3.68 [c]

[a] [HepOH] = 4.00x10^{-1} M, [$PhXn^+$] = 9.66 x 10^{-4} M. For analysis, 80 microliters of reaction solution at different times was injected into 1.92mL acetonitrile containing 0.80M HClO4. Absorbance decay at 473 nm due to $PhXn^+$ with time was determined for kinetic measurements.; [b] Numbers in parentheses are the pooled standard deviations S(pooled), except otherwise noted; [c] Two times of measurements, within 0.6% error in rates.

Table S9. The temperature effects on the rate constants and 1° KIEs of the hydride transfer reaction from 4-heptanol (HepOH) to $PhXn^+BF_4^-$ in acetonitrile [a,b]

Temp (°C)	k_{2H} ($M^{-1}s^{-1}$)	No. of Measurements	k_{2D} ($M^{-1}s^{-1}$)	No. of Measurements	1° KIE
67.0	1.33 (0.00_2)x10^{-4}	3	4.49(0.02)x10^{-4}	3	2.97(0.02)
60.0	8.48(0.25)x10^{-5}	4	2.90(0.04)x10^{-5}	4	2.92(0.10)
54.0	6.46x10^{-5} [c]	2	2.10x10^{-5} [c]	2	3.08 [c]
45.0	3.24(0.03)x10^{-5}	4	9.74(0.41)x10^{-6}	4	3.33(0.14)
36.0	1.62(0.01)x10^{-5}	4	5.24(0.16)x10^{-6}	4	3.10(0.09)
29.0	9.65x10^{-6} [c]	2	2.75x10^{-6} [c]	2	3.51 [c]
22.0	5.63x10^{-6} [c]	2	1.70x10^{-6} [c]	2	3.31 [c]

[a] [HepOH] = 4.00x10^{-1} M, [$PhXn^+$] = 9.66 x 10^{-4} M. For analysis, 80 microliters of reaction solution at different times was injected into 1.92mL acetonitrile containing 0.80M HClO4. Absorbance decay at 473 nm due to $PhXn^+$ with time was determined for kinetic measurements.; [b] Numbers in parentheses are the pooled standard deviations S(pooled), except otherwise noted; [c] Two times of measurements, within 0.2% error in rates.

Table S10. The temperature effects on the rate constants and 1° KIEs of the hydride transfer reaction from DMPBIH to $PA^+BF_4^-$ in acetonitrile [a,b]

Temp (°C)	k_{2H} ($M^{-1}s^{-1}$)	k_{2D} ($M^{-1}s^{-1}$)	1° KIE
55.0	4.40(0.04)x10^2	1.35(0.02)x10^2	3.26(0.05)
45.0	2.94(0.02)x10^2	8.82(0.07)x10	3.34(0.03)
35.0	1.93(0.01)x10^2	5.81(0.04)x10	3.33(0.03)
25.0	1.28(0.01)x10^2	3.85(0.03)x10	3.32(0.03)
15.0	8.63(0.07)x10	2.66(0.03)x10	3.25(0.04)

[a] Repeated on three different days with 6 repetitions each day. Numbers in parentheses are the pooled standard deviations S(pooled). [b] [DMPBIH] = 0.006 M, [PA^+] = 0.0003 M. Absorbance decay at 436 nm due to PA^+ was followed for kinetic measurements.

Table S11. The temperature effects on the rate constants and 1° KIEs of the hydride transfer reaction from DMPBIH to $HA^+BF_4^-$ in acetonitrile [a,b]

Temp (°C)	k_{2H} ($M^{-1}s^{-1}$)	k_{2D} ($M^{-1}s^{-1}$)	1° KIE
55.0	4.77(0.05)x10^2	1.42(0.01)x10^2	3.36(0.05)
45.0	3.18(0.02)x10^2	9.24(0.08)x10	3.44(0.04)
35.0	2.07(0.02)x10^2	6.06(0.05)x10	3.41(0.04)
25.0	1.36(0.01)x10^2	4.02(0.03)x10	3.39(0.03)
15.0	9.03(0.05)x10	2.69(0.02)x10	3.36(0.04)

[a] Repeated on three different days with 6 repetitions each day. Numbers in parentheses are the pooled standard deviations S(pooled). [b] [DMPBIH] = 0.006 M, [HA^+] = 0.0003 M. Absorbance decay at 436 nm due to HA^+ was followed for kinetic measurements.

Table S12. The temperature effects on the rate constants and 1° KIEs of the hydride transfer reaction from DMPBIH to $BA^+BF_4^-$ in acetonitrile [a,b]

Temp (°C)	k_{2H} ($M^{-1}s^{-1}$)	k_{2D} ($M^{-1}s^{-1}$)	1° KIE
45.0	1.23(0.01)x10^3	4.24(0.03)x10^2	2.91(0.04)
35.0	8.54(0.08)x10^2	2.83(0.02)x10^2	3.02(0.04)
25.0	5.78(0.04)x10^2	1.87(0.02)x10^2	3.10(0.04)
15.0	3.84(0.03)x10^2	1.22(0.01)x10^2	3.16(0.03)
5.0	2.55(0.02)x10^2	8.01(0.09)x10	3.19(0.04)

[a] Repeated on five different days with 6 repetitions each day. Numbers in parentheses are the pooled standard deviations S(pooled). [b] [DMPBIH] = 0.006 M, [BA^+] = 0.0003 M. Absorbance decay at 436 nm due to BA^+ was followed for kinetic measurements.

Organic synthesis

Synthesis of N-propylacridinium iodide (PA^+I^-)

7.13g (39.7mmol) of acridine was added to a 75mL high-pressure reaction vessel along with 25mL of HPLC grade acetonitrile. To the reaction mixture 5.75mL (58.4 mmol) of 1-iodopropane was added. The reaction flask was stirred in an oil bath at 120 °C. After 3 days, the product mixture was vacuum filtered and the filter cake was rapidly washed with small amount of cold acetonitrile to give reddish crude product. The crude product was recrystallized by acetonitrile/ether for three times to give red crystals.

PA^+I^-: M.p. 240°C-242°C; ^{1}H NMR (ppm, CD_3CN) δ 9.86 (s, 1H), 8.55-8.52 (m, 4H), 8.43-8.38 (m, 2H), 8.00-7.97 (m, 2H),5.24-5.20 (m,2H), 2.23-2.14 (m,2H),1.26-1.22 (t, 3H).

Synthesis of N-hexylacridinium iodide (HA^+I^-) and N-benzyl acridinium bromide (BA^+Br^-)

2.02 g of acridine (11.2 mmol) in a 100 mL pear shaped flask with a stir magnet was heated in an oil of 130˚C to melt. 3.2 mL (21.7 mmol) of 1-iodohexane or 2.5 mL (21.1 mmol) of benzyl bromide was added dropwise. Stirring the mixture at 130˚C for 30 minutes. Upon cooling, 50 mL of anhydrous diethyl ether was added to rinse the product. The resulting brownish (HA^+I^-) or greenish (BA^+Br^-) product was filtered off. The HA^+I^- product was recrystallized twice with acetonitrile/ether and the BA^+Br^- product twice with methanol.

HA^+I^-: M.p. 155°C-158°C. ^{1}H NMR (ppm, $CDCl_3$) δ 10.45 (s, 1H), 8.74-8.72 (d, 2H), 8.59-8.56 (d, 2H), 8.43-8.39 (m, 2H), 7.95-7.91 (m,2H),5.51-5.46 (m,2H), 2.22-2.13 (m,2H) 1.79-1.72 (m,2H), 1.48-1.37 (m,4H),0.94-0.91 (t, 3H).

BA^+Br^-: M.p.: 197-200°C. ^{1}H NMR δ (ppm, CD_3CN): 10.00 (1H, s), 8.61-8.58 (2H, d), 8.41-8.33 (4H, m), 8.02-7.98 (2H, m), 7.38-7.36 (3H, t), 7.17-7.15 (2H, m), 6.55 (2H, s).

Synthesis of N-propylacridine (PAH), N-hexylacridine (HAH), and N-benzylacridine (BAH)

These compounds were synthesized by the reduction of corresponding 10-alkyl acridinium salts using $NaBH_4$ in methanol. The procedure is the same as the one described in our previous publication for the synthesis of the N-methylacridine (MAH).[1] The PAH and BAH products were recrystallized three times with 95% ethanol. The HAH product was isolated as pale white oil. The compound was directly used to prepare the $HA^+BF_4^-$ salt (see below). Note that the HAH was not used for the kinetic studies, so further purification was not carried out.

PAH: M.p. 68°C-70°C. ^{1}H NMR (ppm, CD_3CN) δ 7.25-7.10 (m, 4H), 6.89-6.85 (m,4H), 3.94 (s, 2H), 3.82-3.78 (m,2H), 1.88-1.78 (m,2H) 1.05-1.01 (t,3H).

BAH: M.p. 110-113°C. ^{1}H NMR δ (ppm, $CDCl_3$): 7.33-7.29 (2H, m), 7.26-7.16 (5H, m), 7.06-7.02 (2H, m), 6.90-6.86 (2H, t), 6.72-6.70 (2H, d), 5.16 (2H, s), 4.04 (2H, s).

Synthesis of the tetrafluoroborate salts of N-propylacridinium ($PA^+BF_4^-$), N-hexylacridinium ($HA^+BF_4^-$), and N-benzylacridinium ($BA^+BF_4^-$)

The salts were prepared by reacting their reduced form (PAH, HAH, BAH) with the tropylium tetrafluoroborate ($Tr^+BF_4^-$) in acetonitrile using our reported method to prepare N-methylacridinium tetrafluoroborate ($MA^+BF_4^-$).[1]

$PA^+BF_4^-$: M.p. 239°C-242°C. ^{1}H NMR (ppm, $CDCl_3$) δ 9.85 (s, 1H), 8.55-8.52 (m, 4H), 8.43-8.38 (m, 2H), 8.01-7.97 (m, 2H), 5.24-5.20 (t, 2H), 2.21-2.13 (m, 2H),1.26-1.22 (t, 3H).

$HA^+BF_4^-$: M.p. 141°C-145°C. ^{1}H NMR (ppm, CD_3CN) δ 9.84 (s, 1H), 8.54-8.50 (m, 4H), 8.424-8.38 (m, 2H), 8.00-7.96 (m, 2H),5.26-5.22 (m, 2H),1.77-1.66 (m, 2H), 1.65-1.46 (m, 2H),1.45-1.33 (m, 4H),0.95-0.92 (t, 3H).

$BA^+BF_4^-$: Melting point: 254°C -257°C. ^{1}H NMR δ (ppm, CD_3CN): 9.99 (1H, s), 8.61-8.58 (2H, d), 8.41-8.33 (4H, m), 8.02-7.98 (2H, m), 7.38-7.36 (3H, t), 7.17-7.15 (2H, m), 6.54 (2H, s).

Synthesis of 9,9'-dideuterated N-propylacridine (PAH-9,9'-d,d) and N-benzylacridine (BAH-9,9'-d,d)

The compounds were synthesized from the reduction of the corresponding 9-acridone by $LiAlD_4$ in dry THF following our reported procedure to synthesize the N-methylacridone.[2] The 10-substituted-9-acridone was prepared from the oxidation of the iodide or bromide salts by KO_2, using the method that we used to synthesize the MAH-9,9'-d,d.[2] The D-content is higher than 98% by NMR.

N-propyl acridone: M.p. 116°C-118°C. ^{1}H NMR (ppm, $CDCl_3$): δ 8.6-8.58 (m,2H), 7.75-7.71 (m,2H), 7.51-7.49 (d, 2H), 7.31-7.25 (m, 2H), 4.34-4.29 (m, 2H), 2.04-1.94 (m, 2H),1.18-1.14 (t, 3H).

N-benzyl acridone: M.P.: 183°C-187°C. ^{1}H NMR δ (ppm, CD_3CN): 8.45 (2H, dd), 7.72-7.67 (2H, m), 7.52-7.50 (2H, d), 7.37-7.27 (5H, m), 7.20-7.23 (2H, d), 5.70 (2H, s).

Synthesis of 4-heptanol, cyclohexanol, 4-heptanol-α-d, and cyclohexanol-α-d

These compounds were synthesized by reduction of 4-heptanone or cyclohexanone with $NaBH_4$ or $NaBD_4$ in methanol using the same procedures of ours to prepare isopropanol-α-d.[3-4]
4-heptanol: ^{1}H NMR (δ, ppm, CD_3Cl): 0.90-1.00 (t, 6H), 1.25-1.55 (m, 9H), 3.60 (m, 1H).
4-heptanol-4-d: ^{1}H NMR (δ, ppm, CD_3Cl) 0.85-1.00 (t, 6H), 1.24-1.58 (m, 9H).

Cyclohexanol: ^{1}H NMR (δ, ppm, CD_3Cl): 3.63 (m, 1H), 1.90 (m, 2H), 1.75 (m, 2H), 1.56 (m, 2H), 1.31 (m, 5H).
Cyclohexanol-α-d: ^{1}H NMR (δ, ppm, CD_3Cl): 1.90 (m, 2H), 1.75 (m, 2H), 1.56 (m, 2H), 1.31 (m, 5H).

<u>Energies and Atom coordinates for the individual molecules in acetonitrile (SMD model)</u>

PhXnH

Electronic energy = -806.787135958 Hartrees, G = -806.561161 Hartrees

H	-0.751110459762	0.429245807492	0.226891904582
H	-1.274124402673	0.963570877538	2.393682303240
C	-1.408916326689	-0.090397280631	2.648992171290
C	-1.210163979729	-1.065113258612	1.668415471336
C	-1.776923425613	-0.454439077513	3.947147633095
C	-1.386483342125	-2.414016892132	2.004279173281
C	-0.801482878585	-0.669322798923	0.253321960172
C	-1.949692198166	-1.797994948243	4.273958160800
H	-1.927559590372	0.317497196648	4.704345023519
C	-1.753239786048	-2.778887485483	3.297391133152
H	-1.236355392831	-3.185472672595	1.242610781033
C	0.565900324297	-1.223815201540	-0.089345850797
C	-1.826864763574	-1.131500181861	-0.760782754515
H	-2.238055715837	-2.083862710368	5.287186268253
H	-1.887513600259	-3.833278863830	3.546201218485
C	1.728100138096	-0.687059993458	0.476648580216
C	0.694523278868	-2.326880003333	-0.937172113391
C	-1.573695784037	-2.238236374425	-1.575205151188
C	-3.070839601081	-0.499762974337	-0.873090134005
H	1.632976126787	0.175029866239	1.142149474096
C	2.979461827202	-1.237354018395	0.216915605915
C	1.943665535726	-2.894015804278	-1.205191502873
O	-0.381505730911	-2.905051132128	-1.549270096806
C	-2.537016942289	-2.717680322396	-2.467675740916
C	-4.038726307078	-0.961801434640	-1.759622493957
H	-3.273698339670	0.368007018965	-0.240031237811
C	3.083261162290	-2.350144221969	-0.623828029843
H	3.872460282539	-0.804081808523	0.669697072683
H	1.996071222537	-3.753288108156	-1.875362157731
C	-3.769206666829	-2.080403338694	-2.554834471509
H	-2.295702435087	-3.585042651993	-3.083791424249
H	-5.001756813033	-0.454320194568	-1.832230140480
H	4.058494275211	-2.793296297045	-0.832675955874
H	-4.521198691274	-2.453616716813	-3.252105679204

$PhXn^+$

Electronic energy = -806.006439251 Hartrees, G = -805.790238 Hartrees

H	1.808821405908	1.178054059426	-1.809659286810
C	2.356421874141	0.667358155354	-1.014955977474
C	1.653500336463	0.000016570246	0.000001722967
C	3.748744358085	0.653552348549	-1.017655950054
C	2.356436682770	-0.667307644768	1.014960622105
C	0.176362086934	0.000000233918	0.000001450533

C	4.445489813959	0.000049558771	0.000003036140
H	4.290860829586	1.159672030590	-1.817827442856
C	3.748758837072	-0.653469262520	1.017661664554
H	1.808847572265	-1.178015484432	1.809664072566
C	-0.548172281258	-1.220194946088	-0.045244913987
C	-0.548197735063	1.220180164893	0.045246328040
H	5.536739135508	0.000062425740	0.000003530920
H	4.290886510030	-1.159575835469	1.817833858301
C	0.062568994240	-2.505750118460	-0.145466311064
C	-1.966883057400	-1.168387799904	-0.030135735284
C	-1.966907304134	1.168343801833	0.030137019248
C	0.062517331743	2.505747839168	0.145465633162
H	1.147653095982	-2.576407723688	-0.208032078467
C	-0.708922347584	-3.640405524565	-0.181013807228
C	-2.754294951046	-2.326139238620	-0.061860213552
O	-2.612232619686	-0.000028686488	0.000000696497
C	-2.754342960121	2.326079105858	0.061860422723
C	-0.708997221434	3.640387471630	0.181012214015
H	1.147600114332	2.576427536859	0.208030457189
C	-2.120592256102	-3.548940091218	-0.128378809779
H	-0.235598505605	-4.619010228238	-0.261451558032
H	-3.839707340987	-2.230327728222	-0.039700882444
C	-2.120665274207	3.548893031344	0.128377842587
H	-3.839753387749	2.230245277704	0.039701025820
H	-0.235693394705	4.619001980460	0.261448493881
H	-2.720000844559	-4.460373367317	-0.154631205629
H	-2.720092497376	4.460314087652	0.154629081412

Cyclohexanol

Electronic energy = -310.721234099 Hartrees, G = -310.582204 Hartrees

C	-1.360447198574	-0.890598830705	-0.483982936739
C	-2.047270115365	-1.506309543997	0.730520925574
O	-2.105039482142	0.261608773298	-0.843795735037
H	-0.330509422597	-0.599539285247	-0.204699288263
C	-3.435888123566	-2.041968367069	0.380531834876
H	-1.413648064428	-2.325998540078	1.107723996659
H	-2.110869746896	-0.748149525451	1.526673569422
H	-1.727748813098	0.615099084519	-1.659864255996
C	-3.366530293614	-3.039227803486	-0.776737497248
H	-3.893464813721	-2.509463582111	1.265790293118
H	-4.079791802873	-1.195540066410	0.091523871846
C	-2.687209935959	-2.418736293662	-1.998285954833
H	-2.789443928007	-3.924662178592	-0.455504972445
H	-4.375320379663	-3.395138131085	-1.037309335852
C	-1.298833945768	-1.887750073715	-1.642508789192
H	-2.614194208419	-3.152661479699	-2.815465601733
H	-3.303296213916	-1.583217649888	-2.369598495214
H	-0.829939267287	-1.402041752523	-2.514823917566
H	-0.640276244106	-2.723102754099	-1.350596711377

Protonated cyclohexanone

Electronic energy = -309.942431217 Hartrees, G = -309.814822 Hartrees

C	-1.633407191801	-0.861236366705	-0.577491622431
C	-2.264578205925	-1.351723668965	0.657791466412
O	-1.133434957218	0.305251876749	-0.552907159974
C	-3.545446588443	-2.153374613754	0.371841993029
H	-1.500205404881	-2.033516598859	1.082019010258
H	-2.403857397029	-0.522237782899	1.361725051266
H	-0.702424735854	0.552177985223	-1.404021413621
C	-3.319441744839	-3.183175954479	-0.729476914247
H	-3.863225004702	-2.630811546684	1.307502195928
H	-4.340632169623	-1.454216390180	0.071935126547
C	-2.839116093905	-2.509697495207	-2.010819203175
H	-2.574612689723	-3.925378567128	-0.397865796655
H	-4.254303003615	-3.726288888493	-0.926823754901
C	-1.541923950561	-1.716498639086	-1.772194037405
H	-2.644808443790	-3.245381251940	-2.801424704122
H	-3.611612669699	-1.819975166517	-2.382696537098
H	-1.219353520705	-1.132835756157	-2.645862686350
H	-0.728393227687	-2.425931174920	-1.520325013462

Isopropanol

Electronic energy = -194.115770071 Hartrees, G = -194.038137 Hartrees

H	0.921641593974	1.736223915549	0.099840960191
O	0.069870733724	1.363025519681	-0.160923578566
C	-0.003961183221	0.048012953879	0.362715553130
H	0.003791242762	0.084646721612	1.469498551042
C	1.178872602176	-0.787923930019	-0.102224455646
C	-1.329419101706	-0.533153872095	-0.090414220940
H	1.125607268353	-1.809785570611	0.301102929377
H	1.191135756164	-0.842816166114	-1.201814712175
H	2.127639522436	-0.340182582433	0.232889609496
H	-2.161693921630	0.101207184875	0.246849443389
H	-1.358714693788	-0.587376303565	-1.189731749735
H	-1.471898819244	-1.544513870760	0.315608670438

Protonated acetone

Electronic energy = -193.339636127 Hartrees, G = -193.274499 Hartrees

H	1.063899191621	1.551575659527	0.425054777618
O	0.124247640507	1.276376890130	0.318973363475
C	0.001430434811	0.056895404256	-0.012832026707
C	1.176766602786	-0.784264905463	-0.251970173354
C	-1.364152444241	-0.453362111872	-0.148920687861
H	0.984257327738	-1.803912095466	0.105944154917
H	1.276555558158	-0.844213036873	-1.351824560798
H	2.093281510584	-0.359795181339	0.175366453646
H	-2.095521918021	0.356859613110	-0.232257574929
H	-1.415629536225	-1.159818518569	-0.987628948496
H	-1.556431367718	-1.034823717442	0.771987222488

Energies and Atom coordinates for the TSes of isopropanol and PhXn$^+$ systems in gas phase

TS1

Electronic energy = -1000.02417162 Hartrees, G = -999.713000 Hartrees

H	1.993174492439	1.269527532248	1.741816692461
O	1.832379903605	0.333808235428	1.953463620979
C	0.619600034994	0.123777955114	2.413844585176
H	-0.169725083192	0.017230560909	1.181452575747
C	-0.104386565100	1.286062582867	3.017412798745
C	0.423110329181	-1.270513796020	2.905598283091
H	-1.160279096101	1.057175607828	3.198153293475
H	0.375689995523	1.513212311275	3.984689793966
H	-0.025252408203	2.175455466017	2.373931398693
H	0.810759681615	-1.990986945345	2.170799578795
H	1.014166523628	-1.380411547850	3.830093657059
H	-0.628810184403	-1.477449315046	3.131432821149
H	-2.281129077925	-0.483947524991	1.793373878641
C	-2.661954943154	-0.176827452252	0.816403927506
C	-1.773362748986	0.123817822541	-0.218013993415
C	-4.039898884365	-0.104508954419	0.610166772271
C	-2.278286498707	0.490780590845	-1.471570099897
C	-0.276068914829	0.004651477993	-0.044788600794
C	-4.536633030748	0.265436935598	-0.637327012616
H	-4.724314096977	-0.338847071775	1.426221189069
C	-3.653472671726	0.560285391747	-1.677062889636
H	-1.589929687406	0.718587867456	-2.288308633292
C	0.326834620203	-1.302622926386	-0.415555377496
C	0.535958686891	1.169964589885	-0.475009350850
H	-5.613221009048	0.323128093481	-0.801130707333
H	-4.038209315183	0.846025391510	-2.656805581359
C	-0.389073637820	-2.510953650230	-0.309968969641
C	1.666152637998	-1.359208541135	-0.829223460771
C	1.860774550874	0.979234346990	-0.903300720938
C	0.046402592468	2.489781747262	-0.388223125361
H	-1.440744626487	-2.477937064458	-0.019692161948
C	0.221128129003	-3.724524630766	-0.579589071332
C	2.290771582908	-2.580609425654	-1.098967272600
O	2.415241499053	-0.244456323651	-1.021705114403
C	2.679128666686	2.069931138696	-1.226071596131
C	0.850689076801	3.571696680358	-0.705868001720
H	-0.988906112022	2.645215916900	-0.076953761806
C	1.569422166439	-3.756426205816	-0.966083406860
H	-0.347312181929	-4.650877174974	-0.498447348830
H	3.332025431517	-2.572424733522	-1.421073239567
C	2.173544949629	3.355973283284	-1.124346472812
H	3.697984081161	1.872202803300	-1.559444859064
H	0.455344682872	4.585259329435	-0.639559811097
H	2.053595919459	-4.710312073400	-1.178981220140
H	2.808996539363	4.204232698724	-1.382195005113

TS3

Electronic energy = -1000.02223819 Hartrees, G = -999.710327 Hartrees

H	-0.247586235225	2.370135322672	2.023603511887
O	0.363075485060	1.750909730604	2.461197002471
C	-0.105151315127	0.522632873227	2.489715635211
H	0.233557984538	0.034069726655	1.152683642977
C	-1.583217328938	0.328014584896	2.419846648325
C	0.727946332306	-0.443476852838	3.266884692678
H	-1.838319799783	-0.713447193225	2.184848072080
H	-1.989657878297	0.565843878694	3.417226851538
H	-2.049012930543	1.005918155984	1.688581049539
H	1.798058372337	-0.261937540939	3.116891435526
H	0.495462976101	-0.293641918902	4.334858142370
H	0.471811468803	-1.476236859230	2.999216875716
H	2.405130028732	0.904888576646	1.312331559161
C	2.680463950358	0.331544046609	0.424555865194
C	1.690361500020	-0.250127759945	-0.374382067521
C	4.026361690587	0.207344626371	0.080885581315
C	2.062178061398	-0.943035212254	-1.532831232267
C	0.218180471839	-0.088397026789	-0.059258748545
C	4.391240414082	-0.489571828348	-1.069212851637
H	4.790394884898	0.660807456601	0.713385243353
C	3.406703848682	-1.058906946405	-1.876749073096
H	1.296386772010	-1.391190702584	-2.168749602494
C	-0.416698840111	1.171001177171	-0.542520821138
C	-0.635380002594	-1.294818485139	-0.239884222392
H	5.443553483972	-0.586904488212	-1.338323207980
H	3.685941074573	-1.599773414587	-2.781770240136
C	0.286631298071	2.390849905312	-0.604870434366
C	-1.773513919393	1.172729394986	-0.907195654126
C	-1.976590543773	-1.155965544282	-0.620910875471
C	-0.156332768731	-2.586569386514	0.050369547404
H	1.352185126147	2.399513227652	-0.370216676170
C	-0.349053683310	3.564411030647	-0.983612255066
C	-2.417713477367	2.350230325513	-1.300077672007
O	-2.531181054761	0.051060079722	-0.897398312925
C	-2.819279573057	-2.266650949878	-0.728678857909
C	-0.985597865731	-3.692055592185	-0.045914324122
H	0.888630059341	-2.706849910180	0.343674037846
C	-1.707802915754	3.540430222243	-1.328851878305
H	0.212959220414	4.497132496042	-1.030835834187
H	-3.469304341967	2.296091795589	-1.582058019290
C	-2.321899704356	-3.527461831605	-0.439587580733
H	-3.851381311842	-2.108353468264	-1.041196550140
H	-0.599017529276	-4.686205183337	0.178054887587
H	-2.209763674938	4.458328306094	-1.637455570035
H	-2.976956809393	-4.395540844289	-0.522654720120

TS2

Electronic energy = -1000.01803807 Hartrees, G = -999.706486 Hartrees

H	1.804094219126	-0.881292499243	2.961707697893

O	0.905667555484	-1.210970137559	2.797598847068
C	0.040630523793	-0.248606625486	2.588961609530
H	0.224568796012	0.019679081631	1.123448675898
C	0.325758383227	1.141259199329	3.066504581886
C	-1.371694122898	-0.712777656505	2.531819610327
H	-0.168483080426	1.881640280302	2.420459481715
H	-0.103332813796	1.239149053580	4.077791441393
H	1.399793269360	1.360034660948	3.122923962075
H	-1.448192633338	-1.686976529652	2.032817719904
H	-1.710048672426	-0.831536972810	3.575095982970
H	-2.015823834352	0.030870865192	2.043187394398
H	2.426796868631	0.713476565051	1.399983126501
C	2.675241136587	0.299815434713	0.420385495778
C	1.657133647667	-0.097299090144	-0.450835373520
C	4.014699490601	0.214885093818	0.040512390712
C	2.000143219234	-0.570557334569	-1.723055307762
C	0.194486691278	0.022793708906	-0.081507768350
C	4.346970346524	-0.264842180210	-1.223963380127
H	4.798252671691	0.530386130984	0.730470070208
C	3.336621733596	-0.651106407790	-2.105332544734
H	1.213871801153	-0.875287827946	-2.416355207027
C	-0.455071187828	1.331421369811	-0.391105430787
C	-0.655008431243	-1.151893709627	-0.416206347758
H	5.392763751864	-0.332921725748	-1.525304141172
H	3.590990089490	-1.017560159903	-3.100627675532
C	0.258670623367	2.544559980042	-0.388393138597
C	-1.833983871279	1.375704737095	-0.646038100044
C	-2.019929600808	-0.972230730716	-0.680758202794
C	-0.154566804380	-2.466335716533	-0.358142878790
H	1.338239893376	2.523887055525	-0.229770282303
C	-0.386730880590	3.751807218652	-0.604583137790
C	-2.492430213765	2.589270594253	-0.865667698989
O	-2.596783260735	0.254756392916	-0.711468994349
C	-2.865395354442	-2.064016132789	-0.900061101723
C	-0.986714531503	-3.554404078483	-0.566417619840
H	0.904089142403	-2.618731276206	-0.141780939699
C	-1.769081179073	3.771453161855	-0.836714454171
H	0.182445785318	4.681306498173	-0.605842207330
H	-3.563360828012	2.569618629553	-1.067642770364
C	-2.346521221176	-3.348278837331	-0.841478937643
H	-3.917447179554	-1.872839436380	-1.111732309756
H	-0.584533181125	-4.566280281605	-0.519432358805
H	-2.280085603562	4.718968709917	-1.011858481024
H	-3.004160153469	-4.201218075011	-1.013707297477

Energies and Atom coordinates for the TSes of cyclohexanol and $PhXn^+$ systems in gas phase

TS10
Electronic energy = -1116.62860540 Hartrees, G = -1116.253749 Hartrees

C	1.885673715814	0.314088896195	-0.150013900678
C	2.333230076439	0.814983304086	1.185934319873

O	1.996937370608	-0.990327853630	-0.281818297084
H	0.449181196641	0.507360546665	-0.033278706482
C	3.868613010372	0.989405628422	1.097489461208
H	1.862863475135	1.786850566349	1.394598206413
H	2.045757722886	0.099279628156	1.969014615725
H	1.872038175178	-1.276601970859	-1.203369488878
C	4.250959868252	1.889020717847	-0.077507315619
H	4.227506818403	1.403176469986	2.049302898364
H	4.328789246105	-0.004049090185	0.980672224043
C	3.714877694783	1.344586429482	-1.401328460947
H	3.852303511236	2.903867437614	0.089119653010
H	5.344022414737	1.987400869481	-0.132840826639
C	2.176893098828	1.170924659574	-1.344521905024
H	3.958459862422	2.012870349810	-2.238051551443
H	4.178756583333	0.369517277430	-1.619968432929
H	1.781250021168	0.706854057991	-2.262097004747
H	1.697095549480	2.152550350989	-1.222738566572
C	-0.691555500767	0.052022810317	0.018400631882
C	-1.527483844438	1.310283402443	-0.052309063162
C	-0.709858176338	-0.673590800067	1.315648208748
C	-0.980818957204	2.580446073995	0.137898156368
C	-0.858740682364	0.003624079864	2.540527559591
C	-1.795078260230	3.713673432831	0.101928384573
C	-0.796328566595	-0.680698693404	3.743076703374
C	-3.162799206578	3.579681198369	-0.121247417377
C	-0.588486191925	-2.068117933930	3.744380443496
C	-3.715243314716	2.311095126123	-0.307749188640
C	-0.456584627297	-2.761230335677	2.551600856707
C	-2.903808024166	1.180752306770	-0.275132485350
C	-0.519834874879	-2.062916197204	1.342368078538
H	-3.338127412213	0.189025344063	-0.419712101421
O	-0.416095334347	-2.806182488597	0.211468307917
H	-4.786715541402	2.201151272575	-0.479243130628
C	-0.574209349097	-2.230891650697	-0.999183225356
H	-3.800978229328	4.463557488874	-0.148544673756
C	-0.540166966870	-3.087511072599	-2.107772870883
H	-1.356933292759	4.701480914428	0.249491003418
C	-0.693793411578	-2.562088093169	-3.380891661671
H	0.089288813362	2.694806928646	0.324763337095
C	-0.888244938254	-1.184039325730	-3.565663159098
C	-0.921558136519	-0.342544327163	-2.465807659889
C	-0.759870738116	-0.848327445611	-1.160538425640
H	-1.082218618839	0.730131809071	-2.595270730976
H	-1.020021780063	-0.778815877509	-4.568930170609
H	-0.673015394593	-3.229573077676	-4.243289408064
H	-0.401991910312	-4.154667979982	-1.933718901132
H	-0.315204978573	-3.841467485562	2.521868953389
H	-0.543290677933	-2.612212738324	4.688604961882
H	-0.918034308148	-0.144809944767	4.684221843255
H	-1.035738978737	1.080906003897	2.528029921826

TS15

Electronic energy = -1116.62793251 Hartrees, G = -1116.253397 Hartrees

C	1.231025012549	-1.263414625018	-0.933790912841
C	1.240936896151	-2.623592931063	-0.307604778820
O	0.813422936766	-1.235539625996	-2.185215465931
H	0.136964568738	-0.667370314468	-0.218789268145
C	2.584579837983	-3.292836793986	-0.682749565509
H	1.174015406291	-2.510372410104	0.785456712512
H	0.387442355422	-3.217029262345	-0.658535140699
H	0.951013077206	-0.364998507637	-2.598340043246
C	3.770000347279	-2.416644268085	-0.285399645661
H	2.630351614197	-4.274175217837	-0.191857081420
H	2.593843020139	-3.471979885814	-1.769170108826
C	3.671009403258	-1.031686721150	-0.920451365567
H	3.804894796999	-2.315570784097	0.812154415328
H	4.709899426220	-2.899289362283	-0.587463478221
C	2.340345601225	-0.343196151313	-0.531973418188
H	4.500051539832	-0.384590329279	-0.604513303978
H	3.725352463059	-1.116014874402	-2.017685651471
H	2.235970780054	0.638318138657	-1.022350903392
H	2.312206004127	-0.196579281287	0.558831865590
C	-0.661145991375	0.183385215144	0.164924703290
C	-1.953402127009	-0.603100061948	0.203558735673
C	-0.574006691235	1.246741802476	-0.875643457928
C	-2.177785638888	-1.683903458525	-0.655300022529
C	-1.213433757788	1.130752713744	-2.126430835696
C	-3.401618127787	-2.352299983152	-0.640717364817
C	-1.047358782805	2.098596987802	-3.106658627167
C	-4.411633048670	-1.940146918026	0.226075623527
C	-0.246769497536	3.220429460200	-2.850238308191
C	-4.196036090457	-0.856239709582	1.077038396275
C	0.374251094126	3.371823597563	-1.620261738543
C	-2.972994068749	-0.189766524405	1.069421237599
C	0.209529276390	2.387835211990	-0.640008261904
H	-2.809275999144	0.658394379088	1.737017581645
O	0.842789131438	2.605054094969	0.536296828260
H	-4.985435351177	-0.525260067628	1.752924028911
C	0.665034275349	1.761877389078	1.583223257126
H	-5.368899741806	-2.462488138395	0.237041195214
C	1.281617426500	2.125179807021	2.784294537102
H	-3.565897876021	-3.195123246957	-1.313088068945
C	1.164468429571	1.293766491467	3.886622007047
H	-1.402724452048	-1.997528623278	-1.357505397177
C	0.433799394004	0.099246673376	3.802211714518
C	-0.181526482885	-0.247894499023	2.610354538149
C	-0.081540630930	0.580283941679	1.476127945516
H	-0.762911841288	-1.169476955828	2.539030246354
H	0.346119598916	-0.551108533731	4.672391727279
H	1.643803563466	1.575243276767	4.825092093434
H	1.841951769742	3.059320254571	2.819558826897
H	0.984961030564	4.242586776211	-1.381562506031

H	-0.121452859469	3.989071381812	-3.613869611825
H	-1.554055138407	1.994356869314	-4.066106062479
H	-1.860763882083	0.272517603712	-2.312530822098

TS5

Electronic energy = -1116.62688863 Hartrees,   G = -1116.251747 Hartrees

C	-0.408995434324	-0.271490263925	2.137561425657
C	-0.300733570554	-1.706737884302	2.572928768863
O	0.514484189962	0.511323640614	2.672275538211
H	0.006393450540	-0.357235910579	0.775632453171
C	-1.337006133846	-2.610057857199	1.911573082177
H	0.726609761518	-2.069691582409	2.439279967511
H	-0.474768937866	-1.667223564213	3.665394966769
H	0.344567300926	1.453085268980	2.499187878912
C	-2.741153659646	-2.033229308485	2.073503532662
H	-1.102665218092	-2.720358545288	0.839667567603
H	-1.265809948839	-3.612460607076	2.353991673681
C	-2.834790314015	-0.662753285058	1.406936133362
H	-3.485547109841	-2.710021233076	1.632767951247
H	-2.983445809902	-1.951932231819	3.146874193900
C	-1.786229844195	0.312699056060	1.955296277215
H	-2.708853007701	-0.785372119399	0.321665820320
H	-3.828135586737	-0.218279705022	1.556958619793
H	-2.071814344005	0.629829542384	2.976683622644
H	-1.725678540436	1.239841830149	1.358131538686
C	0.450391486139	0.058520736569	-0.344224323090
C	1.671163255997	-0.812989027796	-0.536549658182
C	0.714304111447	1.504632092218	-0.119822484330
C	2.477112042005	-1.198199639702	0.542306195746
C	1.906301319916	1.982178311853	0.467353448964
C	3.630791551479	-1.950806047000	0.329304790732
C	2.092031510230	3.332356340466	0.715757728691
C	4.001137353244	-2.311787388868	-0.964855158594
C	1.092037240186	4.251832183168	0.365945301706
C	3.217860215673	-1.908698125930	-2.045694139308
C	-0.075159786013	3.819053400844	-0.242051443953
C	2.059055464275	-1.165115330733	-1.835906638823
C	-0.256733630031	2.454174949833	-0.488072285062
H	1.456587011936	-0.850032928726	-2.689435363942
O	-1.408613384403	2.108898186352	-1.106123556936
H	3.510038369889	-2.172916947601	-3.062749271703
C	-1.608328211238	0.835680300218	-1.527912262084
H	4.904911622623	-2.898610581500	-1.132812429679
C	-2.773731688746	0.605375774235	-2.266864301047
H	4.246185279140	-2.248084202435	1.179317793186
C	-3.053499573695	-0.676481548982	-2.709670765115
H	2.222552942732	-0.885962711328	1.556394642988
C	-2.172936548555	-1.733198795062	-2.427672198665
C	-1.019715796614	-1.493008636281	-1.701061944871
C	-0.712356572342	-0.200552626356	-1.232836008098
H	-0.333802498753	-2.312062407258	-1.478376858686

H	-2.395529175724	-2.740002441820	-2.780950567841
H	-3.961751098982	-0.861864650545	-3.284369241637
H	-3.433151073453	1.447923087491	-2.474561127263
H	-0.858426367369	4.509515004229	-0.554576229797
H	1.238865168528	5.316353613189	0.553058323264
H	3.021481973053	3.678980528224	1.167301730271
H	2.694586244481	1.272884288696	0.717395290774

TS3

Electronic energy = -1116.62563643 Hartrees, G = -1116.250050 Hartrees

C	-0.750258574201	-0.197967073362	-2.042767544196
C	-2.098009925914	0.312274327850	-1.592685921772
O	0.034574247240	0.659462559938	-2.682027773148
H	-0.114849153785	-0.366240208721	-0.809377201766
C	-2.990022688008	-0.747038171874	-0.937839611183
H	-1.973288936432	1.203301176561	-0.954050837895
H	-2.572238997005	0.672257871087	-2.525412260416
H	-0.145720027960	1.579531509858	-2.428844211980
C	-3.002279567161	-2.046787153109	-1.739083664904
H	-2.640075615710	-0.958314483937	0.083926771709
H	-4.003765496954	-0.334918108732	-0.840997074206
C	-1.581886473122	-2.583984957599	-1.905041723422
H	-3.632773554008	-2.790030467903	-1.232718287788
H	-3.450513215798	-1.875853050402	-2.732469670453
C	-0.706292025846	-1.576891476139	-2.646909133994
H	-1.162614034483	-2.795827009639	-0.906112028083
H	-1.579493970449	-3.534107105064	-2.456041062918
H	-1.100560291462	-1.430106430789	-3.669972577442
H	0.338619385445	-1.893201964676	-2.772065234462
C	0.515808819512	0.061374753675	0.273585459709
C	1.739880525784	-0.823431982223	0.296053853326
C	-0.489751125535	-0.184475357414	1.334540774530
C	1.764578131309	-2.096056972816	-0.279160630029
C	-0.673266113457	-1.452287015010	1.919033882614
C	2.895084562684	-2.904459166894	-0.164285396459
C	-1.687170743928	-1.671226863541	2.835407678447
C	4.008920367021	-2.451336396469	0.538429609941
C	-2.540739892110	-0.617732795214	3.200464743744
C	3.985967213644	-1.188127265524	1.129885612536
C	-2.362638668208	0.647598316489	2.665500748811
C	2.861423312325	-0.376381210910	1.009701658771
C	-1.335642642101	0.859223024844	1.740858571158
H	2.850639238487	0.608122756189	1.481235179403
O	-1.199575306571	2.125079171268	1.270918901010
H	4.849661652820	-0.830506704745	1.691855268278
C	-0.154085081520	2.453912939194	0.482124242441
H	4.892708346268	-3.083603238096	0.629346735903
C	-0.007280617914	3.811302160209	0.174480518976
H	2.900721137663	-3.893050629740	-0.624650377490
C	1.050032346882	4.214646189310	-0.624974357534
H	0.892923396733	-2.471903907981	-0.813058800315

C	1.969762428776	3.276779124971	-1.122832502495
C	1.817298413186	1.935698730863	-0.818124535030
C	0.745145493074	1.490658263295	-0.010831998086
H	2.519423909292	1.199139433810	-1.210018728966
H	2.801293129378	3.604009810135	-1.746656184177
H	1.170368622808	5.273097780123	-0.860370993461
H	-0.726815483281	4.518253342015	0.587114887891
H	-2.991492387599	1.491389890870	2.949346019136
H	-3.338543805071	-0.788227314200	3.924305020678
H	-1.815884863491	-2.657825908150	3.280344985874
H	-0.000656401251	-2.265265741683	1.642399199186

TS16
Electronic energy = -1116.62546242 Hartrees, G = -1116.251814 Hartrees

C	1.408838906596	-0.971044938410	-0.967611925007
C	1.432704367198	-2.474316193526	-0.962416965153
O	1.246589948579	-0.466573978303	-2.183749841903
H	0.215867409947	-0.659870442537	-0.318442573310
C	2.848337882017	-2.917361148149	-1.392320417140
H	1.227940004361	-2.835655918375	0.056439058797
H	0.669999220876	-2.862148125470	-1.650396275779
H	1.426834915302	0.487666392291	-2.211031795360
C	3.914168248632	-2.296348942466	-0.493090389604
H	2.893829520683	-4.014380129195	-1.364441817372
H	3.007605782018	-2.611249141800	-2.437868843503
C	3.817081983936	-0.772648808548	-0.489693941305
H	3.795451888250	-2.674513605262	0.536191247650
H	4.914691123365	-2.605162827293	-0.826733275640
C	2.406617905463	-0.305355870090	-0.062500977997
H	4.553388510052	-0.326865497613	0.192471567116
H	4.031541348903	-0.382514395254	-1.497616345838
H	2.322628606929	0.792128523507	-0.122233948792
H	2.212606621163	-0.606500728987	0.978434262039
C	-0.707095825439	0.126229254574	0.129560349220
C	-1.949296513118	-0.707223988806	-0.083515765536
C	-0.563072224542	1.322581247493	-0.726626490843
C	-1.915897880644	-2.101834690265	-0.151212982978
C	-1.075243530002	1.367922720988	-2.041517458827
C	-3.096776654135	-2.832192103567	-0.282478131737
C	-0.860030795552	2.470338826419	-2.850933360284
C	-4.322137085289	-2.172730122688	-0.337563130191
C	-0.123568858797	3.561048379852	-2.361960236841
C	-4.363182245239	-0.780277458343	-0.259879753371
C	0.389083696613	3.546583989669	-1.074305801299
C	-3.185268611668	-0.048655721960	-0.135611570713
C	0.167535947694	2.429388889506	-0.261327477617
H	-3.224475532994	1.040531917131	-0.072574927179
O	0.693968411336	2.475075137411	0.982892588709
H	-5.319759193505	-0.257594419066	-0.296501574781
C	0.425924323916	1.495130524185	1.882080002093
H	-5.246512442795	-2.742334016716	-0.439449009480

C	0.897297813720	1.699776453730	3.181615865869
H	-3.056122427993	-3.920400089189	-0.340413598094
C	0.654681097547	0.738247979895	4.149486380843
H	-0.964837491339	-2.630618785899	-0.094570372397
C	-0.067543395709	-0.422617889349	3.833953057503
C	-0.535490516093	-0.614904877775	2.544975854578
C	-0.287158086664	0.336490832792	1.536962431047
H	-1.115106489889	-1.505940207406	2.299917109640
H	-0.268183430223	-1.167438254724	4.603884693122
H	1.019282673451	0.894191731187	5.165549904337
H	1.439477839329	2.619423041932	3.400944703434
H	0.952349114281	4.384717951424	-0.664101982840
H	0.043762275158	4.432969589213	-2.995803341461
H	-1.261589816676	2.490816279891	-3.863809857960
H	-1.639824339007	0.511777653945	-2.413656917867

TS4

Electronic energy = -1116.62438601 Hartrees,   G = -1116.249519 Hartrees

C	0.331665427503	0.175288174739	2.228530796982
C	1.573810004571	1.003955737790	2.035692527443
O	-0.743051026693	0.803995533537	2.687291146605
H	-0.015755266796	-0.140998248615	0.904543225747
C	2.754722584860	0.227174267222	1.454112586393
H	1.348984347104	1.915819510810	1.458637239529
H	1.818596850468	1.340096609495	3.061780842701
H	-0.709071492341	1.751950365525	2.480550295002
C	2.976885545218	-1.092151314300	2.190714275967
H	2.579169305705	0.024190031049	0.387344215445
H	3.649971115396	0.862161531536	1.500348710998
C	1.709232433266	-1.943681031532	2.172197312229
H	3.809117633667	-1.641122963161	1.729720195826
H	3.269719080502	-0.889804248714	3.234811472686
C	0.538150917153	-1.199237246028	2.813877650249
H	1.458047199169	-2.211730575993	1.132058018340
H	1.864981590738	-2.888261246096	2.710182867308
H	0.752931610618	-1.004378251726	3.881924515870
H	-0.393521392462	-1.777960703378	2.798392897477
C	-0.494729214255	0.021426904546	-0.296050682459
C	-1.983839929205	-0.152612842322	-0.118052366323
C	0.196450279922	-1.043791322509	-1.064858683326
C	-2.551853926166	-0.912701497009	0.906806700837
C	-0.262450672818	-2.374376556891	-1.090191658550
C	-3.930885217465	-1.109881986394	0.961268729240
C	0.443461892068	-3.354170108754	-1.767408442214
C	-4.754959349590	-0.557143059245	-0.015899129228
C	1.624459337087	-3.021929095029	-2.448188898088
C	-4.195212625382	0.195306236166	-1.048660353478
C	2.084790010562	-1.714615588351	-2.460727855747
C	-2.819710198523	0.400977424413	-1.099455498400
C	1.366340232528	-0.732843195909	-1.774115470612
H	-2.387790647756	0.982989305534	-1.915982446324

O	1.863777721244	0.530010446942	-1.829996363761
H	-4.833143857521	0.623254989779	-1.822798726045
C	1.162830192261	1.568206508752	-1.324920795210
H	-5.833471124265	-0.713450453334	0.024953323014
C	1.696709387684	2.846095059891	-1.530522940753
H	-4.360214461027	-1.698956256730	1.772288185140
C	1.023504519365	3.951549678166	-1.036898135526
H	-1.922069561512	-1.353255670536	1.676656756314
C	-0.191468509669	3.800092503346	-0.348197977105
C	-0.713499198114	2.533153832909	-0.148093123862
C	-0.037365015846	1.388368690665	-0.621253224877
H	-1.671795975233	2.404151064869	0.360215099910
H	-0.726754300373	4.677672242635	0.014639690959
H	1.436705925982	4.948037729844	-1.198654459566
H	2.631289706006	2.935713011689	-2.084379623626
H	2.985522992482	-1.421962876956	-3.000077932083
H	2.178672646845	-3.792309605183	-2.985769988711
H	0.076105176993	-4.380188245807	-1.779536964299
H	-1.188740703953	-2.625484201345	-0.571952538038

TS14

Electronic energy = -1116.62413806 Hartrees, G = -1116.250835 Hartrees

C	-1.123227031718	-1.183745295637	-1.257223230456
C	-2.173624640018	-0.127438310672	-1.155122738564
O	-0.522246463775	-1.232160411218	-2.427584000602
H	-0.101289208595	-0.586681106680	-0.395403072233
C	-3.507332049837	-0.738752896153	-1.642492634429
H	-2.276462847690	0.173856058393	-0.099874125845
H	-1.889071073965	0.747003281059	-1.755618458213
H	0.040509363463	-2.018097844702	-2.520980283271
C	-3.849783195104	-1.997877697628	-0.851689158497
H	-4.291090336825	0.023846004312	-1.543374807255
H	-3.417744460633	-0.974623309384	-2.714476876223
C	-2.732644161594	-3.030771809185	-0.961969987870
H	-4.012477845747	-1.738916942929	0.207860856026
H	-4.791348898131	-2.429182214425	-1.219447375956
C	-1.376520143886	-2.448993201970	-0.489968372802
H	-2.948329209647	-3.924867447512	-0.361524018137
H	-2.632229568609	-3.360561205351	-2.008534691624
H	-0.569739600998	-3.181132304690	-0.641622680099
H	-1.425778430237	-2.208712475817	0.584277617019
C	0.659688241042	0.182573476005	0.162310534615
C	2.015229577682	-0.356994716224	-0.235302153633
C	0.280642416114	0.023009911304	1.597474516815
C	2.200479679374	-1.695463677194	-0.593039666631
C	0.739271759853	-1.050004886953	2.384277774276
C	3.468946031658	-2.180781254994	-0.910498626920
C	0.314521333527	-1.206801482857	3.694120485123
C	4.568633292401	-1.326966724040	-0.865984321683
C	-0.580248431915	-0.283154697929	4.252681605133
C	4.394366632772	0.007937063018	-0.499391305407

C	-1.033858122134	0.792253700317	3.505982899307
C	3.126886070169	0.492955576879	-0.187601600346
C	-0.601228802950	0.942553708166	2.185315873341
H	2.996137533682	1.538307498987	0.098417425270
O	-1.069461018030	2.028098022443	1.520070236697
H	5.253469336316	0.678503794928	-0.455812977386
C	-0.581499311943	2.347195857890	0.296833580588
H	5.562571310063	-1.701675670811	-1.112781765954
C	-1.022004565926	3.556424589230	-0.248968957770
H	3.598552777142	-3.228206726042	-1.185452871648
C	-0.592555354088	3.927872472219	-1.513298980900
H	1.351141029947	-2.381930248605	-0.593196073329
C	0.277222269795	3.101360718160	-2.239797044746
C	0.714463230435	1.908087906745	-1.687811936583
C	0.296565181580	1.508605558020	-0.403760317610
H	1.387664009600	1.258855438731	-2.249790780320
H	0.610504721312	3.397218523899	-3.234388889942
H	-0.934470009194	4.871268933445	-1.940990482160
H	-1.696026163964	4.177516246549	0.340900960270
H	-1.712960699094	1.539104195964	3.917212689361
H	-0.912454930747	-0.399331018953	5.284952163510
H	0.684452715248	-2.039924812411	4.291565076983
H	1.454252063818	-1.754158145702	1.955618970709

TS1

Electronic energy = -1116.62382640 Hartrees,   G = -1116.249366 Hartrees

C	-0.171103409173	1.005924291451	1.856328061376
C	-1.208167159561	0.446592297898	2.796652250597
O	1.063185482991	0.673864389874	2.205013023260
H	-0.379747825600	0.224477191085	0.675816610772
C	-2.647240501202	0.836458251884	2.451551250348
H	-1.059115056871	-0.639347228854	2.902053969636
H	-0.928566749855	0.878415948337	3.775952539187
H	1.730455866086	1.227455718790	1.765907586246
C	-2.769779658848	2.317230737679	2.100146652838
H	-3.006695848916	0.247804125525	1.594861793122
H	-3.291717522960	0.578135067847	3.302731947730
C	-1.839450580676	2.662618821257	0.939279407893
H	-3.809962624030	2.552224397173	1.837357247140
H	-2.515947629314	2.938952741455	2.975384106737
C	-0.386383859321	2.394655391093	1.320961576486
H	-2.107339008994	2.053319434479	0.059228030293
H	-1.943350531641	3.714199361952	0.640006909048
H	-0.092962723979	3.057949452743	2.157220602858
H	0.314184873097	2.606784369398	0.496654838060
C	0.078179333680	-0.397953634116	-0.384989556870
C	-1.144057036949	-1.103405969769	-0.922631965216
C	1.135411048043	-1.276581423286	0.167012694193
C	-2.134938833706	-1.608511274275	-0.076074455528
C	0.872329810274	-2.560816046962	0.686304439711
C	-3.209368772153	-2.331273095074	-0.591120921104

C	1.883321925638	-3.324846831949	1.241836796372
C	-3.294525031231	-2.570306436020	-1.961689583556
C	3.194870391085	-2.828393225798	1.288811267372
C	-2.296268692016	-2.092907857459	-2.810205977788
C	3.489233563529	-1.578068382660	0.771560383338
C	-1.225623534287	-1.364755724378	-2.296699065072
C	2.461549321729	-0.814379597288	0.215100383901
H	-0.442465693668	-1.006253350139	-2.966887644433
O	2.814042948252	0.406445196581	-0.265692708737
H	-2.347388937834	-2.290285713949	-3.881571306463
C	1.945816837493	1.118254087547	-1.028099731565
H	-4.133980798631	-3.136073899296	-2.367409579921
C	2.459387433458	2.263564132432	-1.645873584563
H	-3.977659609446	-2.713603048750	0.081974816703
C	1.621936122628	3.045885408342	-2.424389464089
H	-2.057611029205	-1.453090833788	1.000393641958
C	0.275095946705	2.690717167242	-2.595273834610
C	-0.223956517607	1.557050928257	-1.976644371806
C	0.602386215374	0.742540366157	-1.175203202637
H	-1.272602547339	1.284205119999	-2.101946656168
H	-0.378227638657	3.306888855830	-3.212987480685
H	2.018392247326	3.937594919283	-2.911552333733
H	3.513307341150	2.503343240459	-1.504594055353
H	4.500130550614	-1.170785833810	0.775550258588
H	3.992142183555	-3.433544081900	1.722076406607
H	1.661884565664	-4.317718914424	1.632984242167
H	-0.138823644702	-2.962580008105	0.627768745359

References

1. Ma, L.; Sakhaee, N.; Jafari, S.; Wilhelm, S.; Rahmani, P.; Lu, Y., Imbalanced Transition States from α-H/D and Remote β-Type N-CH/D Secondary Kinetic Isotope Effects on the NADH/NAD+ Analogues in Their Hydride Tunneling Reactions in Solution. *J. Org. Chem.* **2019,** *84*, 5431–5439.
2. Maharjan, B.; Raghibi Boroujeni, M.; Lefton, J.; White, O. R.; Razzaghi, M.; Hammann, B. A.; Derakhshani-Molayousefi, M.; Eilers, J. E.; Lu, Y., Steric Effects on the Primary Isotope Dependence of Secondary Kinetic Isotope Effects in Hydride Transfer Reactions in Solution: Caused by the Isotopically Different Tunneling Ready State Conformations? *J. Am. Chem. Soc.* **2015,** *137*, 6653 - 6661.
3. Lu, Y., Qu, F., Moore, B., Endicott, D., Kuester, W. , Hydride Reduction of NAD+ Analogues by Isopropyl Alcohol: Kinetics, Deuterium Isotope Effects and Mechanism. *J. Org. Chem.* **2008,** *73*, 4763-4770.
4. Lu, Y., Qu, F., Zhao, Y., Small, A. M., Bradshaw, J., Moore, B., Kinetics of the hydride reduction of an NAD(+) analogue by isopropyl alcohol in aqueous and acetonitrile solutions: solvent effects, deuterium isotope effects, and mechanism. *J. Org. Chem.* **2009,** *74* (17), 6503-10.
